# An automated framework for localization, segmentation and super-resolution reconstruction of fetal brain MRI

**DOI:** 10.1016/j.neuroimage.2019.116324

**Published:** 2020-02-01

**Authors:** Michael Ebner, Guotai Wang, Wenqi Li, Michael Aertsen, Premal A. Patel, Rosalind Aughwane, Andrew Melbourne, Tom Doel, Steven Dymarkowski, Paolo De Coppi, Anna L. David, Jan Deprest, Sébastien Ourselin, Tom Vercauteren

**Affiliations:** a Wellcome / EPSRC Centre for Interventional and Surgical Sciences, University College London, London, UK; b School of Biomedical Engineering and Imaging Sciences, King's College London, London, UK; c School of Mechanical and Electrical Engineering, University of Electronic Science and Technology of China, Chengdu, China; d Nvidia, Cambridge, UK; e Department of Radiology, University Hospitals KU Leuven, Leuven, Belgium; f Department of Radiology, Great Ormond Street Hospital for Children, London, UK; g Institute for Women's Health, University College London, London, UK; h Medical Physics and Biomedical Engineering, University College London, London, UK; i Institute of Child Health, University College London, London, UK; j Department of Obstetrics and Gynaecology, University Hospitals KU Leuven, Leuven, Belgium

**Keywords:** Fetal MRI, Deep learning, Super resolution, Convolutional neural network, Brain localization, Segmentation

## Abstract

High-resolution volume reconstruction from multiple motion-corrupted stacks of 2D slices plays an increasing role for fetal brain Magnetic Resonance Imaging (MRI) studies. Currently existing reconstruction methods are time-consuming and often require user interactions to localize and extract the brain from several stacks of 2D slices. We propose a fully automatic framework for fetal brain reconstruction that consists of four stages: 1) fetal brain localization based on a coarse segmentation by a Convolutional Neural Network (CNN), 2) fine segmentation by another CNN trained with a multi-scale loss function, 3) novel, single-parameter outlier-robust super-resolution reconstruction, and 4) fast and automatic high-resolution visualization in standard anatomical space suitable for pathological brains. We validated our framework with images from fetuses with normal brains and with variable degrees of ventriculomegaly associated with open spina bifida, a congenital malformation affecting also the brain. Experiments show that each step of our proposed pipeline outperforms state-of-the-art methods in both segmentation and reconstruction comparisons including expert-reader quality assessments. The reconstruction results of our proposed method compare favorably with those obtained by manual, labor-intensive brain segmentation, which unlocks the potential use of automatic fetal brain reconstruction studies in clinical practice.

## 1 Introduction

Fetal Magnetic Resonance Imaging (MRI) has become increasingly important in prenatal diagnosis as a complementary tool to ultrasound, for its advantages in demonstrating pathologies in soft tissues, that may not be apparent or cannot be accurately characterized on prenatal ultrasonography. To mitigate the effect of fetal (and maternal) motion, fast imaging methods such as Single-Shot Fast Spin Echo (SSFSE) are used to acquire thick, low-resolution stacks of 2D slices that can largely freeze in-plane motion (Saleem, 2014). With motion commonly occurring in between slice acquisitions, this generally results in motion-corrupted stacks of slices in multiple orientations with poor 3D image integrity and resolution. In order to assess and quantify fetal brain development and pathology, it is highly desirable to reconstruct a single isotropic, high-resolution volume of the fetal brain in standard anatomical planes from multiple low-resolution stacks acquired in different views.

Currently existing reconstruction toolkits generally rely on an approach that iteratively operates motion correction and super-resolution reconstruction (SRR) (Rousseau et al., 2006; Gholipour et al., 2010; Kuklisova-Murgasova et al., 2012; Kainz et al., 2015b). Since the position and orientation of the fetal brain vary significantly between different patients in relation to maternal structures, localizing the fetal brain and obtaining a segmented mask to exclude the surrounding tissues is crucial to achieve accurate motion correction. Current motion-correction approaches typically employ rigid registration with the assumption that the brain has rigid and surrounding tissues non-rigid motion patterns. Thus, localization and segmentation can help to clearly delineate the brain region so that rigid motion correction becomes meaningful. At present, this usually requires manual localization of the fetal brain and uses manual or semi-automatic methods to obtain fetal brain masks, which is laborious and time consuming.

**Fig. 1:**
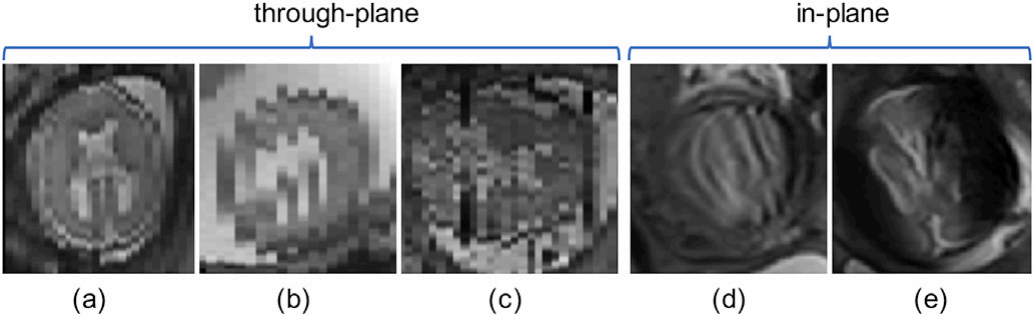
Three example stacks of MRI of fetuses with spinal bifida (a)–(c) with gestational ages of 24, 24 and 29 weeks, respectively. (a) Has a consistent appearance with small inter-slice motion. (b) Shows motion between two interleaved sub-stacks. (c) Illustrates artifact-affected slices with two such ‘outlier’ slices shown in (d) and (e).

With localized or segmented fetal brain masks, multiple motion-corrupted stacks of low-resolution 2D slices can be reconstructed into a single high-resolution 3D brain volume. There are two main challenges associated with the high-resolution volume reconstruction step. First, the inter-slice motion can lead to inconsistent appearance in neighboring slices. This is mainly due to the fact that the SSFSE sequence acquires fetal MR images in an interleaved fashion to reduce the scan time and avoid slice cross-talk artifacts (Gholipour et al., 2014). An *M*-interleaved scanning leads to *M* sub-stacks that are temporally sequential but spatially interleaved, where *M* is usually set as 2 or 3. The motion pattern within each sub-stack is relatively consistent and smooth while that between sub-stacks can be inconsistent, as shown in Fig. 1(b). Moreover, motion during image acquisition can lead to various types of artifacts such as in-plane image blur, slice crosstalk and spin-history artifacts that can considerably affect the image quality of individual slices (Gholipour et al., 2014) as visualized in Fig. 1(c)–(e). Second, robustness against outlier slices characterized by either misregistration or image artifacts is key for a high-fidelity high-resolution reconstruction framework (Gholipour et al., 2010; Kuklisova-Murgasova et al., 2012). However, for previously presented approaches, no complete outlier slice rejection is achieved in Gholipour et al. (2010), and the method of Kuklisova-Murgasova et al. (2012); Kainz et al. (2015b) relies on multiple hyperparameters to be tuned in order to achieve optimal reconstructions while both require time-consuming optimization methods due to their resulting non-convex problem formulation.

**Fig. 2:**
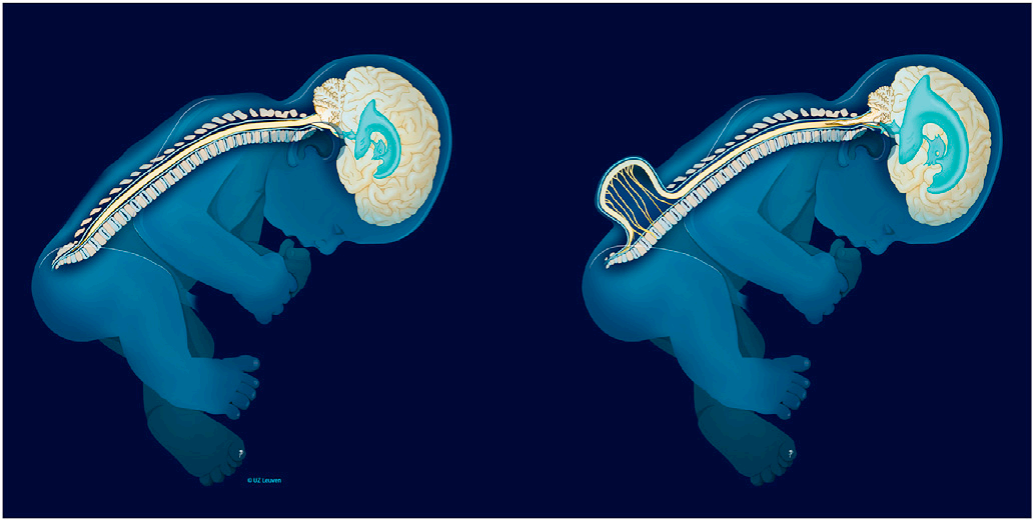
Comparison of a normal fetus and a fetus with open spina bifida showing a Chiari II malformation with ventriculomegaly. Image courtesy of UZ Leuven.

Previous reconstruction frameworks have shown the potential benefit of estimating high-resolution 3D visualizations of the fetal brain (Rousseau et al., 2006; Gholipour et al., 2010; Kuklisova-Murgasova et al., 2012; Kainz et al., 2015b). However, a larger cohort study has not yet been performed to demonstrate their effectiveness on pathological cases where high-resolution 3D brain MRI reconstructions could add important information for more accurate diagnosis and clinical management. One indication for fetal MRI is spina bifida, where MRI plays a role in characterizing the spinal lesion as well as the associated brain changes (Ovaere et al., 2015; Aertsen et al., 2019). In open spina bifida (myelomeningocoele and myeloschisis), a fault in the development of the spinal cord and surrounding vertebrae leaves a gap in the spine, allowing the spinal cord and nerve tissue to bulge through a defect on the fetus's back. Because of a suction gradient by leakage of cerebrospinal fluid at the lesion, the hindbrain descends through the base of the skull where the spinal cord exits (a condition termed a Chiari II malformation). This may be associated with excessive accumulation of fluid in the brain ventricles (ventriculomegaly), as shown in Fig. 2. In these cases, high-resolution 3D reconstructions would aid more accurate measurements, currently performed on low-resolution 2D stacks (Aertsen et al., 2019), and help characterize associated brain changes, ruling out those that are prognostically important.

Furthermore, high-resolution visualization of the brain in standard radiological anatomical planes is highly desirable for clinical assessment. The availability of orthogonal planes of the brain in axial, coronal and sagittal views are of utmost importance for the quality of the examination but are difficult to achieve for fetal MRI in practice due to unpredictable fetal motion (Prayer et al., 2017). True orthogonal planes are vital for adequate measurements and anatomical recognition which improves the detection of pathology, and prevents potential left-right confusion of hemispheres. It also facilitates cross-sectional comparisons of different patient cohorts, and can be especially useful for longitudinal comparisons to visualize anatomical differences of the brain associated with either natural changes or clinical intervention, such as surgery. Thus, template-space reconstructions help to make use of the obtained high-resolution 3D brain visualizations in an optimal way. However, previously presented methods for automatic brain reconstruction in a template space were designed for normal, or mildly pathological, cases only (Tourbier et al., 2017; Gholipour et al., 2017) and suffer from low robustness when dealing with pathological brains such as encountered with spina bifida.

Hence, we hypothesize that a fully automatic reconstruction pipeline based on *automatic* fetal brain localization, segmentation and *robust* reconstruction and template-space alignment steps is favorable to achieve efficient and accurate fetal brain reconstructions for potential clinical translation.

This work is a substantial extension of our preliminary conference publication (Ebner et al., 2018), where we presented a novel framework for automatic fetal brain localization, segmentation and reconstruction in the *standard radiological* anatomical planes. We proposed a novel CNN-based fetal brain segmentation method in a coarse-to-fine fashion to reduce false positives and applied the proposed framework for high-resolution volume reconstruction of MR images of fetuses with spina bifida. Additionally, we introduced an approach for effective complete outlier rejection as alternative to the methods in Gholipour et al. (2010); Kuklisova-Murgasova et al. (2012), that relies on a single hyperparameter only and retains a linear least-squares formulation which can be solved efficiently.

In this paper, we give a more detailed description of the framework and make substantial extensions in three aspects. First, we propose an additional fast template space alignment method that is robust to large brain morphology changes such as encountered in spina bifida. Second, we further show the superiority of our automatic localization, segmentation and reconstruction methods by comparing them with different variants and state-of-the-arts. Third, we validate the proposed framework with a larger dataset of images from both normal fetuses and fetuses with spina bifida (Aertsen et al., 2019). Experimental results show that our framework can achieve comparable reconstruction output to that of manual segmentation-based reconstruction, and it outperforms existing fetal brain extraction and reconstruction methods on different cohorts of fetuses.

## 2 Related works

### 2.1 Fetal brain localization and segmentation

Extracting fetal brain from fetal MRI usually serves as a prerequisite step for high-resolution volume reconstruction of the fetal brain. Anquez et al. (2009) proposed an automated fetal brain segmentation method by localizing the fetal eyes and then segmenting the neighboring skull bone content, which can lead to a poor performance when the inter-slice motion is large. Taleb et al. (2013) used a template-based method to generate fetal brain masks. It obtains a region of interest (ROI) based on the intersection of multiple scans of the same patient, and then registers the ROI to an age-specific template. Tourbier et al. (2017) used template-to-slice block matching and deformable slice-to-template registration for automatic fetal brain localization and segmentation. It achieved good performance at the cost of a very long computational time up to several hours. Kainz et al. (2014) proposed to localize the fetal brain by voxel classification using rotation invariant volume descriptors. Keraudren et al. (2013) used bundled Scale-Invariant Feature Transform (SIFT) features to fit a 3D bounding box of the fetal brain where prior knowledge of the fetal brain development was used to define size and shape constraints for robust localization. Keraudren et al. (2014) extended that method for fetal brain segmentation with image-specific online learning based on Random Forests. It is limited by hand-crafted features and inefficiency during inference. Additionally, some deep learning-based object detection methods such as R-CNN (Girshick et al., 2014) and YOLO (Redmon et al., 2016) are designed for detection of thousands of objects from natural 2D RGB images. However, applying them to fetal brain localization from 3D MRI needs further investigation.

Recently, deep learning with CNNs has been used for fetal brain segmentation from fetal MRI. Rajchl et al. (2016) used a fully convolutional neural network (FCN) for fetal brain segmentation under distributed weak supervision. Salehi et al. (2018) used a 2D U-Net (Ronneberger et al., 2015) for slice-by-slice fetal brain segmentation. These methods predict the fetal brain mask directly without a localization step and, compared with previous methods, are more efficient at test time in terms of computational time. However, they can easily cause false positives and show poor performance for challenging cases.

### 2.2 Fetal brain reconstruction

Rousseau et al. (2006) proposed a slice-to-volume registration (SVR) method for fetal brain reconstruction based on semi-automatic segmentation results. It consisted of three steps: motion correction, volume reconstruction and contrast correction. In the motion correction step, each low-resolution stack is globally aligned first followed by a hierarchical slice package motion correction approach based on the temporal slice interleave. Iterative reconstructions are used as reference for motion correction which were obtained by using scattered interpolation with a narrow Gaussian kernel as the point spread function (PSF). A contrast correction step is used to correct the local relative intensity distortion between the low-resolution stacks. Jiang et al. (2007) used multilevel scattered B-spline interpolation for the reconstruction task that requires sufficient samples to allow full representation of the structure to be reconstructed. Kim et al. (2010) proposed a reconstruction-free registration approach that relies on a slice intersection motion correction (SIMC) method that directly co-aligns multiple stacks of 2D slices which was followed by a single Gaussian-weighted averaging step for the volumetric reconstruction. Gholipour et al. (2010) formulated the volumetric reconstruction step as a super-resolution reconstruction problem that allowed a minimum error representation of the obtained high-resolution volume, whereby a slice acquisition model was used. For the super-resolution problem, it used a robust M-estimation formulation that minimizes a Huber's error function to reduce the influence of potential outliers. Kuklisova-Murgasova et al. (2012) built on this idea and proposed a reconstruction method with *complete* outlier rejection that can entirely exclude identified misregistered or corrupted voxels and slices using expectation-maximization (EM)-based robust statistics. Additional intensity matching was used to compensate inconsistent scaling factors and bias fields of acquired slices. Kainz et al. (2015b) developed a GPU-accelerated implementation of Kuklisova-Murgasova et al. (2012), and proposed to automatically select the stack with least motion as the reference stack. Tourbier et al. (2017) proposed a fully automated reconstruction pipeline including template-space alignment step for the high-resolution visualization in the standard radiological anatomical planes, but presented gestational age-matching to select the template from the normal brain atlas for healthy and mildly pathological cases only. Hou et al. (2017) utilized CNNs to predict the initial transformation parameters of SVR in the motion correction step. McDonagh et al. (2017) proposed a context-sensitive upsampling method based on CNNs to improve the resolution of each low-resolution stack, and then used the upsampled low-resolution stacks as the inputs of an SVR-based 3D reconstruction method. Alansary et al. (2017) proposed a patch-to-volume registration (PVR) framework to reconstruct the whole uterus by splitting each slice into smaller patches used for rigid motion correction. However, apart from the much higher computational requirements, this leads to overall non-rigid motion correction and, thus, suboptimal outcomes for rigidly moving regions such as the fetal brain. Hou et al. (2017, 2018) utilized CNNs to predict the initial transformation parameters of SVR in the motion correction step to achieve more robust initialisations for the slice-to-volume registration step.

Thus, the existing methods have either focused on automatic segmentation of the fetal brain without demonstrating their utility in the context of automatic fetal brain reconstruction for a larger patient cohort, or investigated the reconstruction problem using manually or semi-automatically segmented fetal brain masks. Moreover, there are no appropriate studies showing the performance of pipelining independently developed methods. Therefore, to the best of our knowledge, existing works have not fully solved all these issues which are vital for a robust, consistent, fully-automated reconstruction framework that allows for clinical translation.

## 3 Methods

**Fig. 3:**
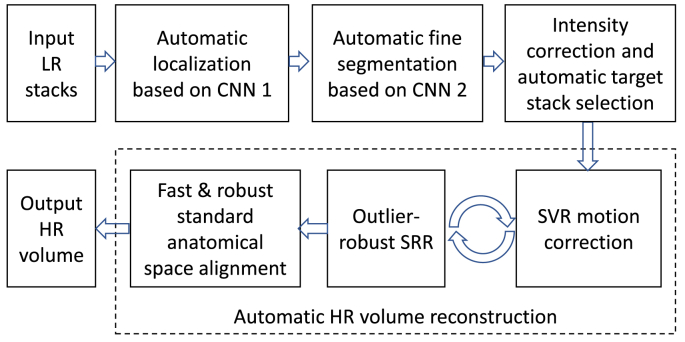
The proposed fully automatic framework for fetal brain MRI reconstruction to obtain high-resolution (HR) visualizations in standard anatomical planes from multiple low-resolution (LR) input stacks. The automatic localization, segmentation and reconstruction parts are detailed in Figs. 4, Figs. 5 and 6, respectively.

An overview of our proposed fully automatic framework for fetal brain reconstruction is depicted in Fig. 3. We first use a CNN to automatically localize the fetal brain region in each input low-resolution stack and obtain a 3D bounding box of the fetal brain. Within the bounding box, we use another CNN to automatically generate a fine segmentation of the fetal brain. The automatic high-resolution volume reconstruction stage includes the two-step iterative SVR and outlier-robust SRR step followed by a fast and robust standard anatomical template space alignment step. For the outlier-robust SRR, we propose a novel outlier rejection method by defining a similarity measurement to remove outlier slices and frame the SRR problem as a linear least-squares formulation that can be solved efficiently. For the template-registration step, we propose a rigid registration approach based on symmetric block-matching between the SRR and a brain-volume-matched template that is initialized by the rigid alignment of the respective principal brain axes (PBA). The three stages of automatic localization, segmentation and reconstruction are detailed in Sections 1, 2, 3.3, respectively. All implementations are available as open-source packages.^2^

**Fig. 4:**
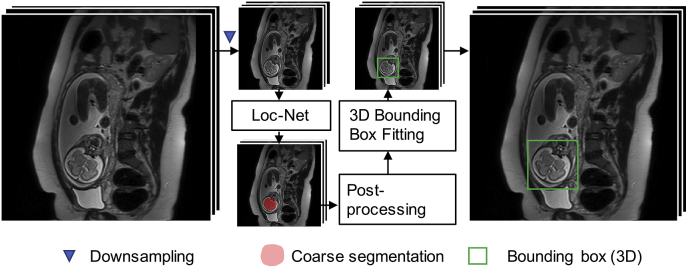
The proposed fetal brain localization method using a CNN (Loc-Net) to obtain a coarse segmentation followed by 3D bounding box fitting.

**Fig. 5:**
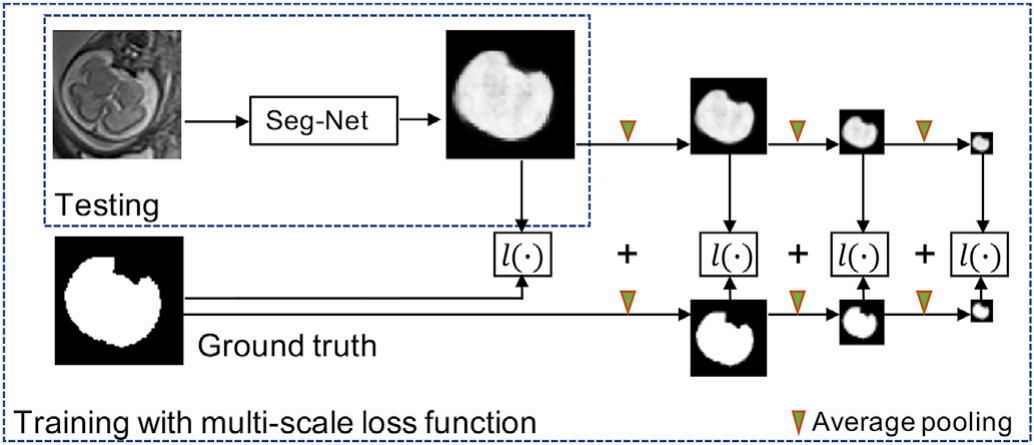
The proposed fetal brain segmentation method using a CNN (Seg-Net) that works on the localization result. We propose to use a multi-scale loss function to train Seg-Net.

**Fig. 6:**
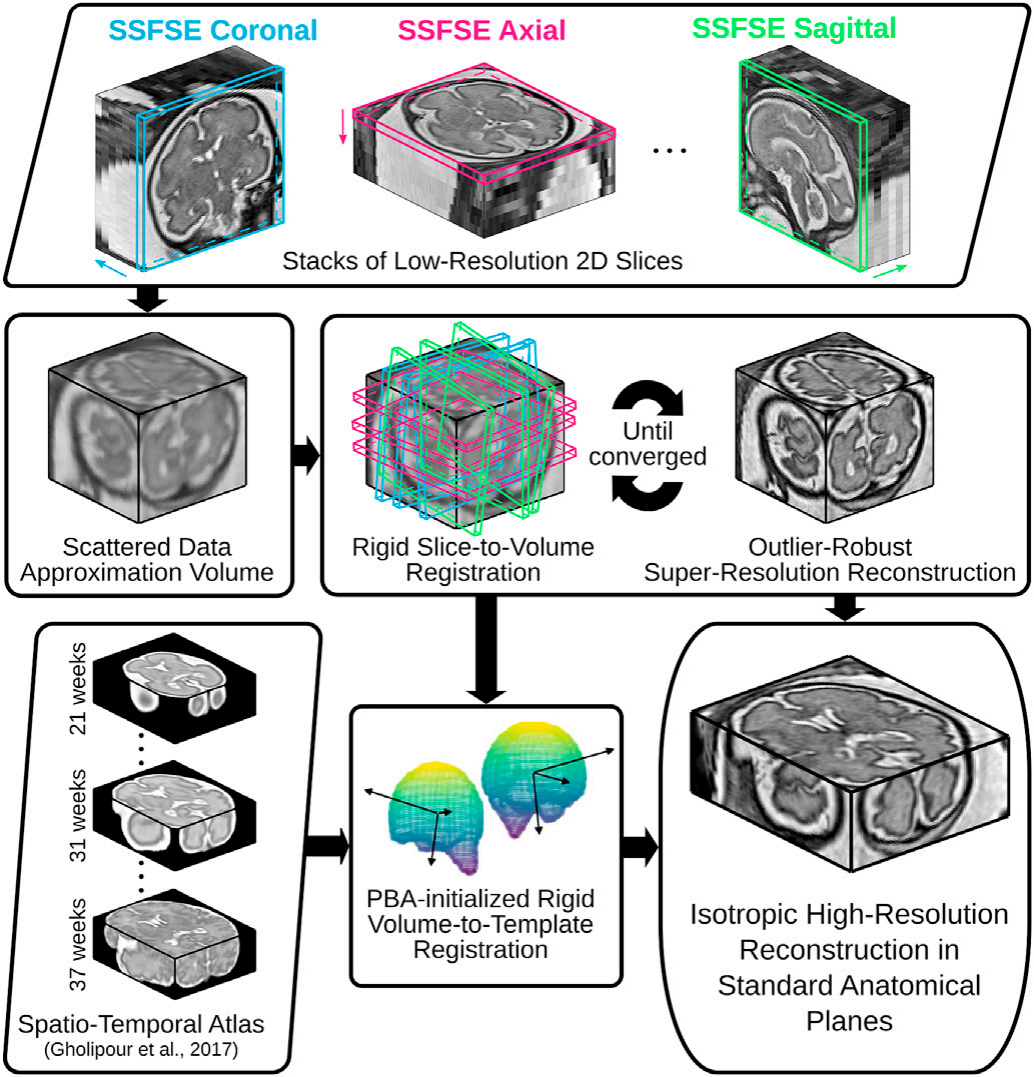
The proposed outlier-robust high-resolution volume reconstruction method for fetal brain MRI. As part of a two-step motion-correction/volumetric reconstruction cycle, we propose an effective robust SRR method for complete outlier rejection that relies on a single hyperparameter only and retains a linear least-squares formulation. A fast template-space alignment, which is robust also for pathological brains, is achieved by using a principal brain axes (PBA)-initialized rigid volume-to-template registration based on symmetric block-matching.

### 3.1 Localization based on coarse segmentation

Differently from traditional top-down object localization methods using sliding window classification (Criminisi et al., 2009) or bounding box regression (Gauriau et al., 2015; He et al., 2017), we use a bottom-up strategy for fetal brain localization based on a coarse segmentation by a CNN with pixel-level prediction. The advantage of such a localization method is that it gives an explainable support for the localization result and is well-suited for single-object localization. To reduce computational requirements, we apply the CNN at a down-sampled version of an input low-resolution stack, as shown in Fig. 4. We refer to this network for the fetal brain localization task as **Loc-Net**.

The proposed framework is theoretically amenable to different CNN models. However, as the input low-resolution stack has a large inter-slice spacing and is potentially corrupted by motion between neighboring slices, it is more compelling to use a 2D CNN than a 3D CNN. We choose the 2D P-Net (Wang et al., 2018) for its compactness and efficiency. It consists of six blocks of convolution layers with dilated convolution (Yu and Koltun, 2016) to preserve resolution for dense prediction. The first five blocks have 2, 2, 3, 3 and 3 convolution layers respectively and they have dilation parameters of 1, 2, 4, 8 and 16, respectively. The convolution channel number for these layers is 64. Multi-scale features from these five blocks are concatenated and fed into the 6-th block which is a voxel-wise classifier with 1 × 1 convolution. A softmax layer is used to obtain probability-like outputs.

Let *I* denote a stack of slices and $I_{i}$ represent the *i*-th 2D slice of *I*. To reduce the inference time and memory consumption for the localization task, we down-sample $I_{i}$ to a given size, i.e., 96×96, obtaining $I{'}_{i}$. We keep the through-plane resolution the same as the input stack, and use I' to denote the stack of down-sampled 2D slices. As shown in Fig. 4, to get a 3D bounding box of the fetal brain in a stack, we first use the Loc-Net to obtain a segmentation of I' by stacking the 2D segmentations, i.e., a coarse segmentation.

With the coarse segmentation of the down-sampled stack I', we employ two post-processing steps to reduce segmentation noise and obtain a smoother result. First, we use a 3D morphological closing and opening operation on the result of Loc-Net. Then we select the largest connected 3D component as the post-processed coarse segmentation of the fetal brain and fit a 3D bounding box to the component as the localization result in I'. The final localization result for input *I* is obtained by rescaling the bounding box to the original space of *I* and expanding by a margin of 5 mm.

### 3.2 Fine segmentation

After the localization, we further obtain a fine segmentation of the fetal brain from *I* with a second CNN that is referred to as **Seg-Net**. It works on the ROI of the localization result of *I* to reduce false positives of the dense prediction. Similar to the localization step, we use the 2D P-Net structure (Wang et al., 2018) for the fine segmentation rather than a 3D network considering the inter-slice spacing and motion.

Due to the change in appearance of the fetal brain at different gestational ages and as a consequence of the presence of pathologies such as spina bifida, it is challenging to achieve robust segmentation results. We propose a multi-scale loss function for training to improve the performance of fine segmentation. The commonly adopted logistic loss and Dice loss functions for image segmentation use a sum of pixel-wise losses (Sudre et al., 2017) and only penalize prediction errors at the finest scale, without considering the relationship between neighboring pixels at a larger scale. This potentially leads to noisy and spatially inconsistent segmentations. In contrast, dealing with the image in the scale-space representation helps to achieve more robust results, as shown by previous works inspired by the scale-space theory (Lindeberg, 1994; Hu et al., 2018).

We propose a training loss function across multiple scales as depicted in Fig. 5. Let *Y* represent the pixel-wise probability prediction of an image given by a segmentation CNN and *G* denote the corresponding pixel-wise probabilistic ground truth. The loss function l(Y,G) measures the similarity between *Y* and *G* and guides the network to obtain a segmentation as close as possible to the ground truth. It is commonly defined as a pixel-wise function for segmentation tasks. For example, the Dice loss function is defined as (Milletari et al., 2016; Sudre et al., 2017):

(1) $$l_{Dice}\left(Y,G\right)=1-\frac{2{\sum^{\;}}_{i}^{N}y_{i}g_{i}}{{\sum^{\;}}_{i}^{N}y_{i}^{2}+{\sum^{\;}}_{i}^{N}g_{i}^{2}}$$

where *N* is the number of pixels. $y_{i}$ represents the probability of pixel *i* being the foreground given by *Y* and $g_{i}$ represents that probability given by *G*. Let *s* be a scale index (s∈{1,2,…}), and $Y_{s}$ and $G_{s}$ be the downscaled versions of *Y* and *G* at scale *s*, respectively. Then the proposed multi-scale loss function is

(2) $$L\left(Y,G\right)=\frac{1}{S}\overset{S}{\underset{s=1}{\sum }}l\left(Y_{s},G_{s}\right),$$

where *S* is the total number of scales. Thus, the loss function L(Y,G) is the average of $l\left(Y_{s},G_{s}\right)$ across multiple scales. When s=1, $Y_{s}$ is the same as *Y*, and when s>1, $Y_{s}$ is a downscaled version of *Y*. $Y_{s}$ can be obtained by down-sampling *Y* or applying max-pooling on *Y*. However, both methods cause the obtained $Y_{s}$ to contain little contextual information. In contrast, Gaussian smoothing and average-pooling summarize the prediction of a local patch for more contextual information. Since average-pooling is more efficient and more straightforward to implement than Gaussian smoothing, we use average-pooling for the downscaling. Let $Pl_{avg}\left(\cdot \right)$ denote the average-pooling operation. We set the pooling kernel size as 2×2 with stride size 2×2. Therefore, $Pl_{avg}\left(\cdot \right)$ averages every neighboring 2×2 pixels. $Pl_{avg}\left(\cdot \right)$ is used recursively to down-scale *Y* and *G* for larger scales *s*:

(3) $$Y_{s}=\{\begin{array}{cc} Y & \text{for ​}s=1\\ Pl_{avg}\left(Y_{s-1}\right) & \text{for ​}s>1 \end{array}$$

With a larger *s*, $Y_{s}$ and $G_{s}$ encode the prediction and the ground truth at a higher level with more non-local information. Therefore, L(Y,G) not only penalizes the pixel-wise difference between *Y* and *G*, but also encourages their similarity at multiple non-local scales. In this paper, we use the Dice loss function as the loss function at each scale due to its good performance in dealing with imbalanced classes, i.e., $l\left(Y_{s},G_{s}\right)=l_{Dice}\left(Y_{s},G_{s}\right)$. We set the total number of scales *S* as 4, as shown in Fig. 5.

### 3.3 Robust high-resolution volume reconstruction framework

The steps of the high-resolution volume reconstruction stage are shown in Fig. 6. We briefly list them here and further detail the main contributions in Sections 3.3.1, 3.3.2. For a set of low-resolution stacks of 2D slices acquired in multiple orientations, we preprocess the images using the bias field correction method (Tustison et al., 2010). Using a volume-to-volume registration based on symmetric block-matching (Modat et al., 2014), all stacks are rigidly aligned with an automatically chosen target stack (more details in 4.2). Based on the brain segmentation of the target stack, all remaining, volumetrically-aligned, stacks are intensity corrected using a linear regression with the masked target stack voxel intensities serving as reference values. An initial high-resolution volume is obtained by applying a scattered data approximation (SDA) scheme on the low-resolution stacks that uses an efficient discrete implementation of Nadaraya-Watson kernel regression (Vercauteren et al., 2006; Ebner et al., 2017). It is based on nearest neighbor sampling onto a regular grid followed by a subsequent Gaussian blurring operation for each single slice. Similarly, SDA is used to obtain a brain mask high-resolution volume from the individual low-resolution stack masks. Then, an updated high-resolution volume is obtained through a two-step iterative registration-reconstruction approach (Rousseau et al., 2006; Gholipour et al., 2010). In each iteration, the rigid registration step registers the slices to the high-resolution volume constructed in the previous iteration for motion correction constrained by the respective slice and high-resolution brain mask. Subsequently, the reconstruction step constructs a high-resolution volume and brain mask from the aligned slices and segmentations, respectively. After reconstruction in the subject's space, we rigidly align the high-resolution volume to a spatiotemporal atlas of normal brains (Gholipour et al., 2017) to obtain the reconstruction in the standard anatomical planes.

#### 3.3.1 Outlier-robust super-resolution reconstruction

After each SVR step of the two-step registration-reconstruction iteration *i*, an SRR step is used to recover the most likely high-resolution volume $x^{i}$ that satisfies the slice acquisition model (Gholipour et al., 2010)

(4) $$y_{k}^{i}=A_{k}^{i}x^{i}+e_{k}^{i}$$

where $y_{k}^{i}$ is the *k*-th slice in a stack. $A_{k}^{i}$ represents the image acquisition process including rigid transformation, slice selection, blurring according to the PSF, and down-sampling. $e_{k}^{i}$ denotes the vector of observed noise. The intensity of each voxel in a low-resolution slice is therefore influenced by a certain neighborhood of this voxel within a high-resolution volume x given by the assumed PSF that is specific to the slice profile of the MR acquisition (Liang and Lauterbur, 2000). For SSFSE sequences, a common approximation is given by a slice-aligned 3D Gaussian function that depends on the in- and through-plane resolution of the low-resolution slice (Jiang et al., 2007; Kuklisova-Murgasova et al., 2012). The position and orientation of the slice (and PSF) within the volume is estimated in the rigid SVR step. In order to prevent misregistered or artifact-corrupted outlier slices from affecting the reconstructed high-resolution volume, we propose a robust SRR with outlier rejection in a maximum a-posteriori (MAP) formulation:

(5) $$x^{i}:=\underset{x\geq 0}{\text{arg}\;\text{min}}\left(\underset{k\in K_{\beta }^{i}}{\sum }\frac{1}{2}{\left\|y_{k}^{i}-A_{k}^{i}x\right\|}_{\ell^{2}}^{2}+\frac{\alpha }{2}{\left||\nabla x|\right|}_{\ell^{2}}^{2}\right)$$

where α≥0 denotes a regularization parameter, ∇ the differential operator, and $K_{\beta }^{i}$ a set of indices of inlier slices

(6) $$K_{\beta }^{i}:=\left\{1\leq k\leq K\;:\;\;\text{Sim}\left(y_{k}^{i},\;A_{k}^{i}x^{i-1}\right)\geq \beta \right\}$$

that demonstrate high agreement with their simulated counterparts projected from the previous SRR iterate using a similarity measure Sim and a threshold β>0. Thus, slices with a value of Sim(⋅) lower than β are regarded as outliers and rejected in the SRR step. More complex SRR models have been proposed in addition to the MAP formulation including modifying (5) to rely on robust M-estimator (Gholipour et al., 2010) and total variation formulations (Tourbier et al., 2015). However, while they substantially increase the computational cost, in our experience, they tend to show little improvement in the obtained reconstruction quality in case of appropriate motion correction of SSFSE-like sequences (Ebner et al., 2019). Assuming a fixed $K_{\beta }^{i}$, we obtain a convex SRR problem with complete outlier rejection in a linear least-squares formulation which we solve using *matrix-free* operations (Diamond and Boyd, 2015; Ebner et al., 2017). We use a dedicated linear least-squares solver to deal with this large linear system whereby positivity is enforced by clipping negative values.

Furthermore, we create an high-resolution brain mask by applying the fast SDA approach on the motion-corrected inlier slice masks which is used for both motion correction and the labelling of inlier-slices in (6) in the subsequent iteration.

#### 3.3.2 Reconstruction in standard radiological anatomical planes

Obtaining the high-resolution fetal brain reconstructions in the standard radiological anatomical planes can facilitate brain studies and is typically favored for the clinical assessment by clinicians. To define the template space, we deployed the recently presented spatiotemporal atlas^3^ (Gholipour et al., 2017) which was constructed from 81 normal fetuses scanned between 19 and 39 weeks of gestation. Rigid registration can be used to align the subject-space SRR to a template. However, given the substantial morphological differences in brain volume and shape between pathological and normal fetuses, a direct registration approach is likely to get stuck in local minima leading to an incorrect template space alignment. To avoid this problem, we propose to use an initialized transformation that is based on the rigid alignment of fetal brain masks only. Using principal component analysis, we first rigidly align the principal brain axes (PBA) of the template and high-resolution brain masks whereby the template is selected based on brain-volume matching. Following the PBA-based initialization, we perform a 3D rigid registration based on block-matching (Modat et al., 2014). For increased robustness, all four permutations of the right-handed bases of principal eigenvectors are tested and the best registration transform is selected as determined by the normalized mutual information similarity between warped SRR and template.

After the 3D rigid registration, we use an additional SRR step to obtain the high-resolution volume in the template space, considering that the resampling process during the 3D rigid registration may affect the image quality.

## 4 Experiments and results

### 4.1 Data

The automatic reconstruction framework was applied to routinely acquired clinical images of fetuses with normal brains, yet scanned for other anomalies, and fetuses with spina bifida (SB) that were scanned at University Hospitals KU Leuven between March 2011 and August 2016 as reported in Aertsen et al. (2019). Access to anonymized images was facilitated through the GIFT-Cloud platform for data sharing (Doel et al., 2017). For normal fetuses, 134 stacks from 37 individuals were scanned at the gestational age (GA) of 27.30±4.11 weeks (“normal” group A). 32 fetuses with spina bifida were scanned before fetal surgical closure at a GA of 23.06±1.64 weeks (“pre-surgery” group B1), and 16 of them were additionally scanned after fetal surgical closure at a GA of 25.69±1.21 weeks (“post-surgery” group B2). Details of the dataset are summarized in Table 1. The distribution of the GAs for the experimental data set is shown in Fig. 8. Due to a local 1.5T scanner upgrade from Siemens Sonata to Siemens Aera in the hospital during the scanning period, two different scanners were involved in our study but the acquisition protocol remained unchanged with identical parameter settings. For each study, 3 to 9 SSFSE stacks in at least three different orientations were collected with pixel size 0.39 mm–1.48 mm and slice thickness 2.50 mm–4.40 mm. All images were acquired with no slice overlap nor gap using an echo time of 133 ms and a repetition time of 1000 ms. For the purpose of testing the robustness of our proposed framework, we used all available SSFSE stack acquisitions and therefore also kept heavily motion- and artifact-corrupted stacks and also images where brains were only partially scanned.

**Table 1:** Information of the datasets used for experiments. The number of available subjects and stacks are listed for training, validation and testing, respectively. Gestational age (GA) is stated as mean and standard deviation.

	Group A	Group B1	Group B2
Pathology	Normal	Spina bifida (pre-surgical)	Spina bifida (post-surgical)
Subjects	(26, 4, 7)	(12, 4, 16)	(0, 0, 16)
Stacks	(78, 12, 44)	(36, 12, 119)	(0, 0, 105)

**Table 2:** Assessment of the robustness of the proposed template-space alignment approach. The comparison shows the number of successful template space alignments based on a total of 39 cases with 7 normal (group A), 16 pre-surgical and 16 post-surgical spina bifida cases (groups B1 and B2). A template space alignment was considered successful if a correct alignment in the standard anatomical planes was confirmed visually. FLIRT is based on correlation ratio as similarity measure whereas NiftyReg uses symmetric block-matching based on NCC. Generally, NiftyReg achieves a more robust alignment given a sufficiently good initial alignment. Using our proposed principal brain axes (PBA)-initialized block-matching registration step, a very robust template-space alignment without a failure case can be achieved even for pathological brains. The SRR (S) with the overlaid SRR (L)/(S)/(M) brain masks for the failed B1 case is shown in Fig. 13.

	SRR (L)			SRR (S)			SRR (M)		
	A	B1	B2	A	B1	B2	A	B1	B2
NiftyReg	0	0	0	1	0	0	1	0	0
FLIRT	0	0	0	4	0	4	3	0	4
FLIRT∘ PBA-init	0	0	0	**7**	0	4	4	0	3
NiftyReg∘ PBA-init	**2**	0	0	**7**	**15**	**15**	**7**	**16**	**16**
Total number of cases	7	16	16	7	16	16	7	16	16

**Fig. 7:**
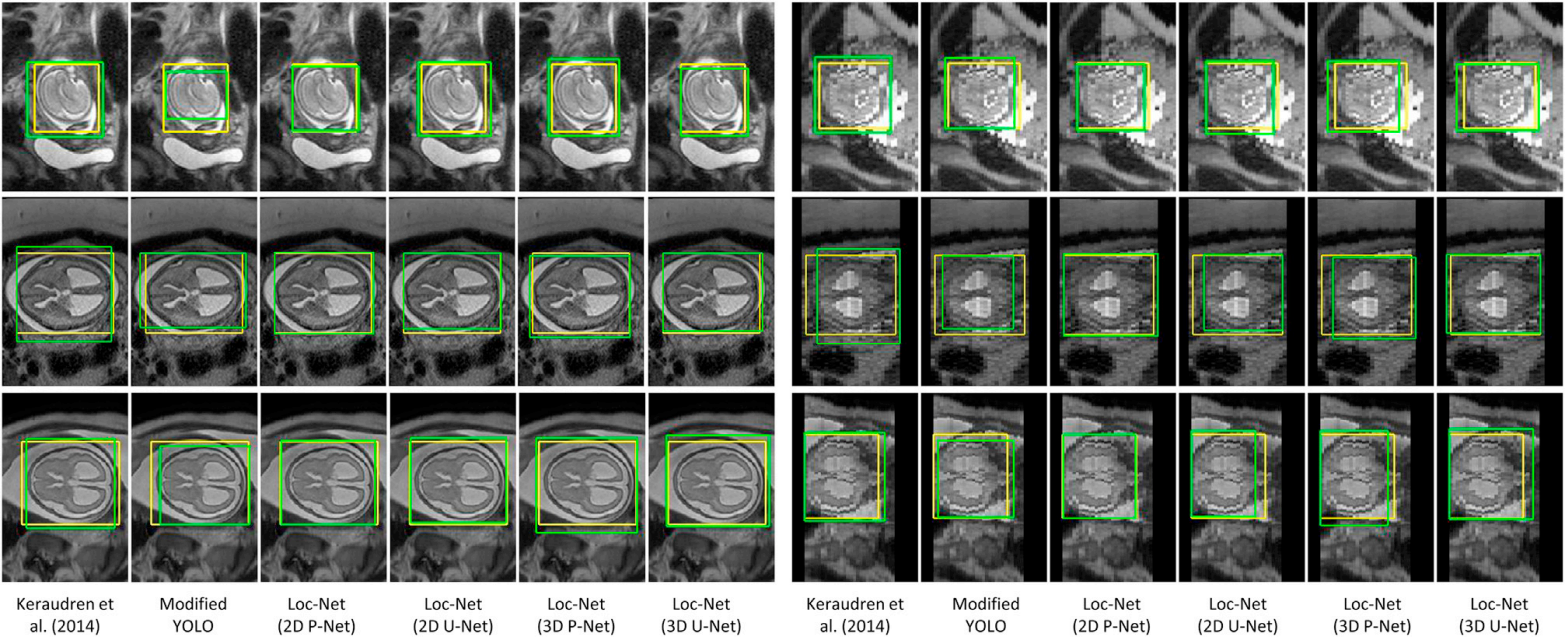
Visual comparison of different methods for fetal brain localization. The three rows show examples from Group A (controls), B1 (pre-surgical spina bifida), and B2 (post-surgical spina bifida), respectively. Column 1–6: in-plane. Column 7–12: through-plane. Yellow: ground truth. Green: detection results.

**Fig. 8:**
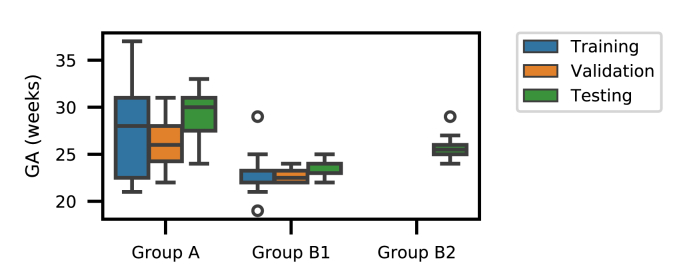
Distribution of gestational age in the experimental fetal image set.

For the fetal brain detection and segmentation set-up, 78 stacks of 26 patients from Group A and 36 stacks of 12 patients from Group B1 were used for training, and 12 stacks of 4 patients from Group A and 12 stacks of 4 patients from Group B1 were used for validation. The remaining images were used for testing, as shown in Table 1. Manual segmentations of the fetal brains on the 2D slices were used as the ground truth for the segmentation task, and the bounding box of the manual segmentation was extended by 5 mm to be used as the ground truth for fetal brain localization. We normalized the intensity of each stack by its mean and standard deviation.

### 4.2 Implementation details

Our CNNs were implemented in TensorFlow^4^ using NiftyNet^5^ (Li et al., 2017; Gibson et al., 2018). For the training of Seg-Net, we set the number of scales *S* to 4 in (2) and employed Dice loss as the loss function used in each individual scale. The detection/segmentation experiments were implemented with an NVIDIA GeForce GTX 980 GPU. For both Loc-Net and Seg-Net, we used Adaptive Moment Estimation (Adam) (Kingma and Ba, 2015) for training, with initial learning rate $10^{-3}$, batch size 10, weight decay $10^{-7}$ and 10k iterations at which point the performance on the validation set stopped to increase.

The high-resolution volume reconstruction algorithm was applied to the testing data of Group A (normal), B1 (pre-surgical spina bifida) and B2 (post-surgical spina bifida) cases using the obtained automatic segmentation results to guide the rigid SVR step. All stacks were preprocessed using the bias field correction method N4ITK described in Tustison et al. (2010) by using the implementation of Insight Toolkit (ITK).^6^ The reconstruction was performed with three cycles of the two-step motion correction and robust volumetric reconstruction iterations, which was found to be sufficient for algorithmic convergence (Inline Supplementary Fig. S3). For automatic target stack selection, we empirically chose the stack with (estimated) brain volume closest to 70% of the median brain volume using the automatically segmented (Seg-Net) brain masks. This approach was chosen for simplicity to define a target stack that shows good brain coverage but avoids stacks with unrealistically high brain volume estimates due to false-positive segmentations or heavy motion-corruption. However, and as shown in the results section, this may still lead to a target stack with substantial motion artifacts suitable to test the robustness of our algorithm also for challenging cases. The orientation of the subject space is defined by the target stack whereby the reconstruction grid is obtained by extending the bounding box of the union of the volumetrically aligned stack masks by 10 mm in each direction. Based on this target stack, all remaining, volumetrically aligned, stacks were intensity corrected using linear regression where the masked target stack voxel intensities served as reference values. For the respective volume-to-volume (and volume-to-template) rigid registrations we deployed the symmetric block-matching algorithm RegAladin that is based on normalized cross-correlation (NCC) as part of NiftyReg^7^ (Modat et al., 2014). The implementation of the SDA approach was based on the Young & Van Vliet recursive Gaussian smoothing filter^8^(Vidal-Migallon et al., 2013). We empirically chose a standard deviation of 1 for both the high-resolution volume and brain mask high-resolution volume SDAs. For the rigid slice-to-volume motion correction steps we used ITK with NCC, whereby both the individual slice mask and the current high-resolution mask volume iterate were used to constrain the registration. Similarly, for the volume-to-template rigid registration we used the final high-resolution mask volume to estimate the PBA-based initialization transform, and to constrain the block-matching-based 3D rigid registration together with the template mask for achieving template-space alignment. Experiments were performed to investigate the sensitivity of the proposed outlier-robust SRR method to the outlier-threshold β and the input fetal brain masks (Inline Supplementary Figs. S1 and S2). By choosing NCC as the similarity measurement function Sim(⋅) in (6), a good balance between conservative slice retention and effective outlier rejection was found for β=0.8. For the experiments, we set the threshold value β to be 0.5,0.65 and 0.8 per iteration to account for increasing accuracy in (5), respectively, whereby the slice similarities were evaluated only for the slice-projected high-resolution mask volume voxels. The matrix-free implementation of the forward operator $A_{k}^{i}$ in (5) (and its adjoint) was done by extending the resampling operator in ITK to allow for oriented Gaussian filtering^9^ representing the oriented PSF kernel whereby the SciPy LSMR algorithm as dedicated linear-least squares solver was used for efficient numerical minimization of (5). The isotropic resolution of the high-resolution volume was set to match the final template space resolution of 0.8 mm (Gholipour et al., 2017) for both subject and template space reconstructions. The regularization parameter α=0.01 was determined by visual assessments supported by L-curve studies (Hansen, 2001). We reconstructed the entire field of view for both subject and template spaces from the brain-motion corrected slices to provide anatomical context beyond the brain.^10^ Our Python code, including both the automatic brain segmentation tool fetal_brain_seg^11^ and the outlier-robust SRR framework NiftyMIC,^12^ is publicly available.

### 4.3 Localization results

For the choice of network structure of Loc-Net, we compared 2D P-Net with 2D U-Net, 3D P-Net (Wang et al., 2018) and 3D U-Net (Çiçek et al., 2016) to investigate whether 2D or 3D networks are more suitable for uncorrected fetal MR image stacks. We also compared our coarse segmentation-based localization method with a modified YOLO (Redmon et al., 2016) that uses a CNN to predict the 2D coordinate and size of a fetal brain bounding box and the associated foreground probability in each slice directly. We employed the CNN structure used by Redmon et al. (2016) and changed the foreground class number to 1 for our task. These networks were all implemented using NiftyNet. In addition, we compared Loc-Net using these network structures with the method described in Keraudren et al. (2014) 13 that is based on classification of image regions using SIFT features and combined with prior knowledge of brain size and shape based on gestational age.

Fig. 7 shows the fetal brain localization results for three cases from Group A, B1 and B2 respectively. In the first case, the centroid of the bounding box obtained by Keraudren et al. (2014) is close to that of the ground truth. However, the size of the localization result is larger than that of the ground truth. In contrast, the result of our Loc-Net with 2D P-Net matches better with the ground truth. It can also be observed that the result of 2D P-Net is better than that of the other three networks. In the second case, the in-plane visualization shows that similar results are achieved by the 2D and 3D networks. However, the through-plane visualization shows that 2D P-Net and 2D U-Net outperform their 3D counterparts.

Quantitative evaluations of these localization methods are shown in Fig. 10. We calculated the Intersection over Union (IoU) score and centroid distance between the localized 3D bounding box and the localization ground truth. Fig. 10 shows that 2D P-Net outperforms 2D U-Net when used as Loc-Net, and both of them achieve better localization accuracy than their 3D counterparts. Our Loc-Net with 2D P-Net achieved average IoUs of 86.54%, 84.74% and 83.67% for these the groups of fetuses, respectively, and it outperformed Keraudren et al. (2014) and the modified YOLO. The stack-level runtime of our proposed localization method was 2.35 ± 1.02s, and the corresponding time of Keraudren et al. (2014) was 15.03 ± 3.54s.

**Fig. 9:**
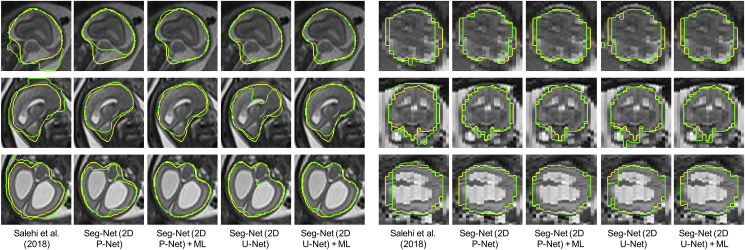
Visual comparison of different methods for fetal brain segmentation. The three rows show examples from Group A (controls), B1 (pre-surgical spina bifida), and B2 (post-surgical spina bifida), respectively. Column 1–5: in-plane. Column 6–10: through-plane. Yellow: ground truth. Green: segmentation results.

**Fig. 10:**
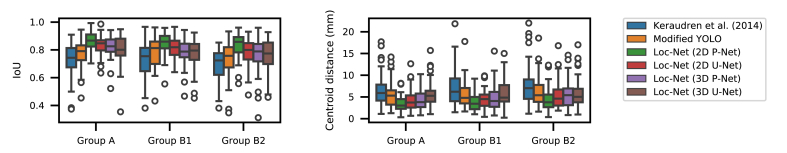
Quantitative evaluation of different methods for fetal brain localization.

### 4.4 Segmentation results

We compared using 2D P-Net and 2D U-Net as the Seg-Net. As a baseline, both of them were trained with the Dice loss with a scale s=1, i.e. at the input resolution. Then we trained these networks with the proposed multi-scale (ML) Dice loss. These four variants are referred to as Seg-Net (2D P-Net), Seg-Net (2D U-Net), Seg-Net (2D P-Net) ​+ ​ML, Seg-Net (2D U-Net) ​+ ​ML respectively. All of them take the same localization result of Loc-Net (2D P-Net) as input for a fair comparison. We also compared them with the method of Salehi et al. (2018) that applies 2D U-Net to the whole input image for segmentation without a localization stage. We followed their implementation available onlin e^14^ and trained the model from scratch with our training images. Fig. 12 shows the effect of the number of scales used in our multi-scale loss function on the segmentation performance. The results are based on validation images from Group A and B1. We found that the segmentation performance was improved when the scale number increased from 1 to 4. Using 5 scales did not lead to additional improvement in the segmentation accuracy. The training of the P-Net using single-scale and multi-scale loss functions took 12k and 10k iterations or early stop when there was no further performance improvement on the validation set, respectively.

**Fig. 11:**
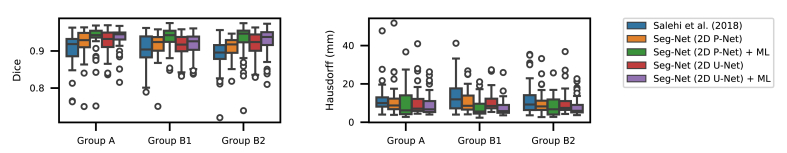
Quantitative evaluation of different methods for fetal brain segmentation.

**Fig. 12:**
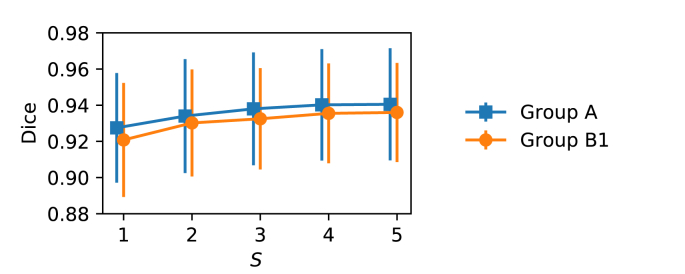
Fetal brain segmentation performance obtained by our multi-scale loss function using different number of scales *S*. The results are based on validation images from Group A and B1.

Fig. 9 presents a visual comparison of the different fetal brain segmentation methods. It shows that the method of Salehi et al. (2018) tends to generate false positives in tissues surrounding the fetal brain. In contrast, the variants of Seg-Net based on localization results achieve more accurate segmentation with reduced false positives. The results of Seg-Net (2D P-Net) and Seg-Net (2D U-Net) have some under-segmentations and unsmoothed contours. By training with the proposed multi-scale loss function, their corresponding results are more spatially consistent and accurate.

Fig. 11 shows quantitative evaluations of these segmentation methods for the fetal brain. We calculated the Dice score and Hausdorff distance between the segmentation results and the pixel-level ground truth. It shows that all the Seg-Net variants outperformed Salehi et al. (2018). Seg-Net (2D P-Net) ​+ ​ML achieved average Dice scores of 93.21%, 93.87% and 92.94% for Group A, B1 and B2 respectively, and it significantly outperformed Seg-Net (2D P-Net) that was trained without ML. The total runtime (forward pass time) of our CNN-based localization and segmentation steps was 3.65s ± 1.34s for one stack including preprocessing and image I/O.

### 4.5 Outlier-robust SRR results

For the experiments, we computed the high-resolution volume reconstructions using various methods: 1) the automatic localization results based on Loc-Net (2D P-Net), 2) the automatic fine segmentation results obtained by Seg-Net (2D P-Net), and 3) manual segmentation results. These three variants are denoted as SRR (L), SRR (S) and SRR (M), respectively. Additionally, we applied the state-of-the-art toolkit (Kainz et al., 2015a)^15^ as described in Kainz et al. (2015b) using the manual segmentations as input masks, denoted as Kainz et al. (M).^16^

**Figure 13:**
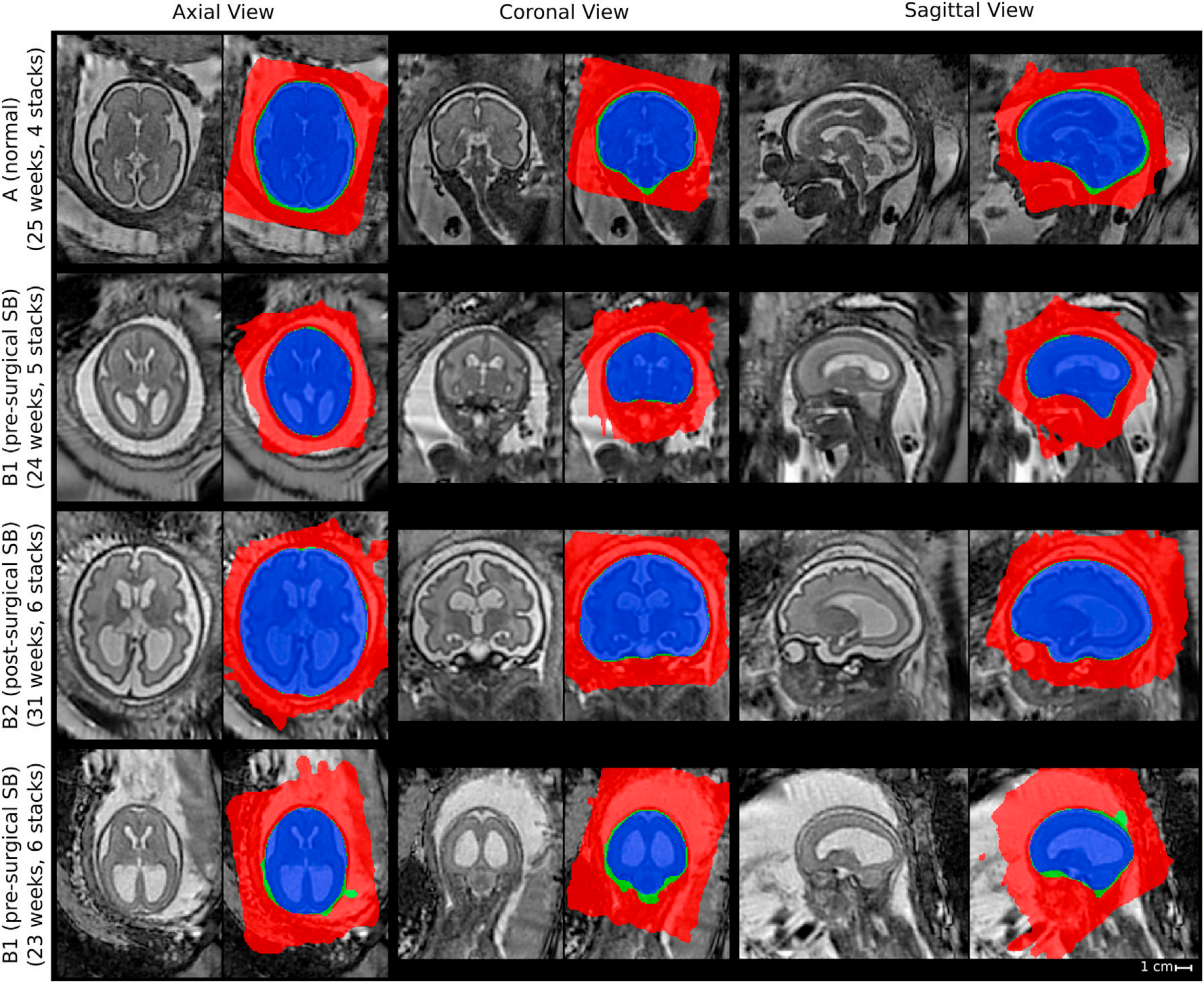
Comparison of SRR (S) with overlaid SRR (L)/(M)/(S) high-resolution masks obtained using either the manual masks (SRR (M); blue colour), the automatic segmentations by Seg-Net (SRR (S); differences to SRR (M) in green colour) or the localization results by Loc-Net (SRR (L); differences to SRR (M)/(S) in red colour). The respective visualizations of SRR (S) were obtained by reconstructing the entire template-space field of view using the brain-motion corrected slice transformations transformed into the template space. The last row shows the only B1-case that failed in the template-space alignment step for SRR (S), see Table 2; the final alignment was obtained after manual re-initialization of the volume-to-template registration.

All of the 39 cases of the groups A (7), B1 (16) and B2 (16) were used for analysis as at least one of the reconstruction methods provided a successful reconstruction in the subject-space. In Table 2 we demonstrate the effectiveness of the proposed template-space alignment step. For comparison purposes, we also provided the number of successful template-space alignments using NiftyReg, FLIRT^17^(Jenkinson et al., 2002) and their respective compositions. Overall, only two cases failed at the template-space alignment step for SRR (S) (one each for B1 and B2) for our proposed method whereas all alignments were successful for SRR (M). All seven cases of group A were successfully reconstructed and aligned. Therefore, the success rate of our proposed framework for all the groups was 37/39 (and 39/39 when the template-space alignment is not considered). The poor performance of SRR (L) for the template space alignment step can be explained by the obtained, rectangular-shaped high-resolution brain volume masks, which leads to non-informative PBA-initialization and volume-to-template registration mask constraints. The failed template-space alignments were manually initialized for SRR (S) so that in total 39 cases were available for the following evaluations. Some visual comparisons of the obtained SRR (S) in the template space along with the high-resolution mask reconstructions for different input masks are provided in Fig. 13.

**Fig. 14:**
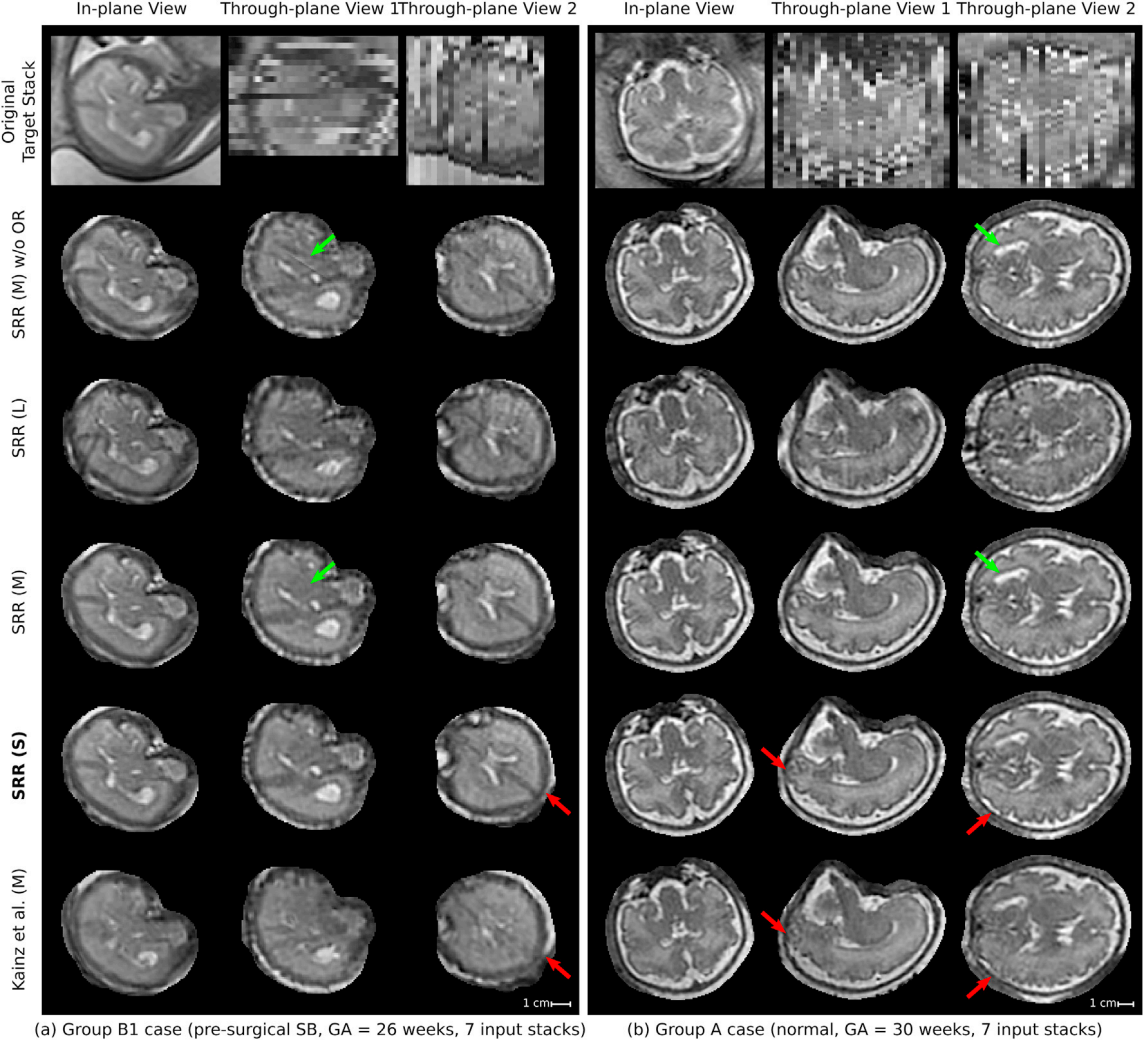
Qualitative comparison of reconstruction methods in the subject space. Visual comparisons of different reconstruction methods for a B1 (left) and an A (right) case where challenging target stacks were (automatically) selected. Additional visualizations associated with the For the group A case (b), additional visualizations are provided to assess the outlier-rejection performance (Fig. 16) and for template space comparisons (Inline Supplementary Fig. S6). Dilated SRR (M) masks were used for visual cropping. SRR (M) without outlier rejection (OR) presents various artifacts. Similarly, the localization masks as used for SRR (L) lead to poor reconstruction outcomes despite the use of outlier rejection. The outlier-robust results SRR (M) and the proposed SRR (S) based on manual and automated brain masks, respectively, provide successful reconstructions and are, visually, almost indistinguishable. Green arrows indicate artifacts in SRR (M) without OR that are eliminated using our proposed OR method. Red arrows show differences between our proposed method and Kainz et al. (M).

Fig. 14 presents a visual comparison of the obtained SRRs for a B1 (pre-surgical spina bifida) and an A (normal) case in the subject space. Despite the challenging target stacks due to intra-stack motion, in-plane blur and intensity artifact corruption, successful reconstructions were obtained using the proposed outlier-robust SRR method. In particular, the high-resolution visualizations for SRR (M) and SRR (S) appear visually almost indistinguishable.

In the supplementary material, additional experiments are summarized to investigate the influence of the intensity correction steps (bias field correction and linear intensity correction) on the obtained reconstruction outcomes (Inline Supplementary Fig. S4). The results underline that both the bias field correction and subsequent linear intensity correction steps lead to statistically significant improvements towards more coherent intensity values of the obtained volumetric reconstructions. Furthermore, we investigated the success rate of the proposed template-space alignment method for different template space selections including the volume-matched template (proposed) and the age-matched template (Tourbier et al., 2017). The results indicate a higher robustness for the proposed, volume-matching approach (Inline Supplementary Table S1).

**Fig. 15:**
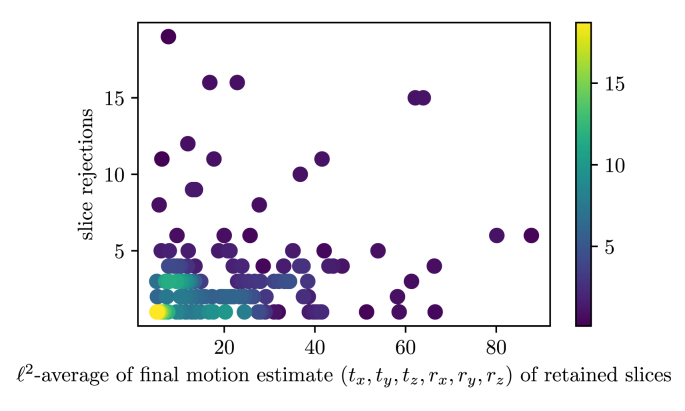
Histogram relating the number of slice rejections with the average slice motion per stack. The mean values of the $\ell^{2}$-norm of translation $t_{x},\;t_{y},\;t_{z}$ (mm) and rotation $r_{x},\;r_{y},\;r_{z}$ (degree) parameters of the non-rejected slices for each individual stack after the final motion correction iteration i=3 for SRR (S) for all 39 cases are shown. The stack associated with the sample in the upper-left corner is shown in Fig. 16.

**Fig. 16:**
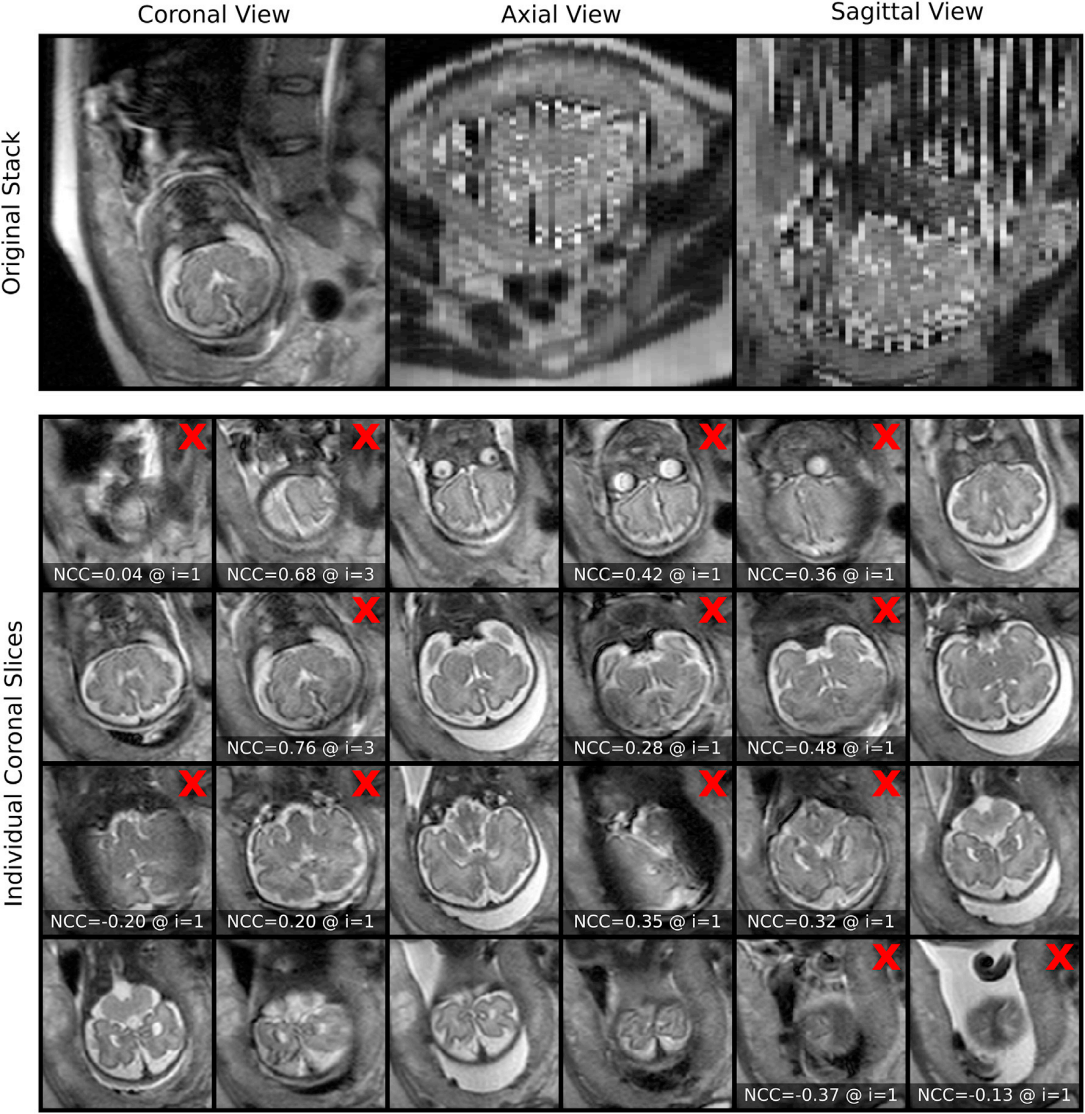
Stack associated with the upper-left corner in Fig. 15 showing substantial in-plane artifacts with relatively moderate slice motion for the non-rejected slices. Red crosses mark the slices that were automatically rejected by the proposed outlier-robust SRR (S) algorithm (only the slices covering the brain are shown; additional six, automatically segmented slices outside the brain were successfully rejected too). The NCC slice similarities $\text{Sim}\left(y_{k}^{i},\;A_{k}^{i}x^{i-1}\right)<\beta_{i}$, at the time of rejection at iteration i∈{1,2,3} with $\left(\beta_{1},\;\beta_{2},\;\beta_{3}\right)=\left(0.5,\;0.65,\;0.8\right)$ are shown in addition. Thus, the outlier-rejection method is able to successfully detect and reject artifact-corrupted slices while keeping slices with good in-plane quality for the final volumetric reconstruction step. It is worth noting that this stack served as the target stack for the SRR algorithm. Successful reconstructions in subject and template spaces for that case are shown in Fig. 14b and Inline Supplementary Fig. S6, respectively.

To investigate the performance of our proposed outlier-rejection method, the number of slice rejections relative to the amount of motion present in the stacks was analyzed (Fig. 15). Whereas relatively few slices are rejected for the majority of stacks with estimated little or moderate slice motion, a higher slice-rejection rate can be observed for stacks associated with higher estimated motion. However, a few stacks show a very high number of slice rejections despite a relatively small average motion of non-rejected slices with the most extreme sample shown in Fig. 16. This comparison underlines that the outlier-rejection method is able to successfully detect and reject artifact-corrupted slices while keeping slices with good in-plane quality for the final volumetric reconstruction step. It is worth noting that the stack in Fig. 16 served as the target stack for the SRR algorithm for that case. Regardless of this poor reference, high-quality reconstructions were obtained with their visualizations in subject and template spaces shown in Fig. 14(b) and Inline Supplementary Fig. S6, respectively.

**Fig. 17:**
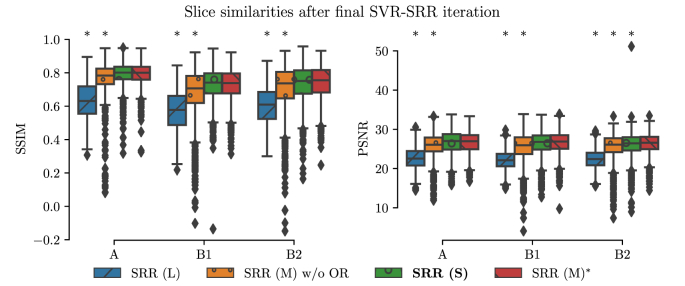
Quantitative comparison of different reconstruction methods based on $\text{Sim}\left(y_{k}^{i},\;A_{k}^{i}x^{i}\right)$ after the final SVR-SRR iteration (i=3) in terms of SSIM and PSNR. A * denotes a significant difference compared to SRR (M) within each group based on Kruskal-Wallis with post-hoc Dunn tests (p<0.05). Thus, SRR (S) and SRR (M) appear of similar volumetric self-consistency as quantified by the similarities between motion-corrected and respectively projected high-resolution volume slices.

For quantitative evaluations of the obtained reconstruction outcomes, we used $\text{Sim}\left(y_{k}^{i},\;A_{k}^{i}x^{i}\right)$ after the final SVR-SRR iteration (i=3) to measure the similarities between the motion-corrected slices of the input low-resolution stacks and their corresponding simulated slices from the reconstructed high-resolution volume. We present structural similarity index measure (Wang et al., 2004) (SSIM) and peak-signal-to-noise ratio (PSNR) measurements for the comparisons here. Alternative similarity measures (NCC; normalized mutual information, NMI; root mean squared error, RMSE; mean absolute error, MAE) were also generated and are presented in Inline Supplementary Fig. S5 for the sake of manuscript conciseness. Fig. 17 illustrates that all methods apart from SRR (S) produce statistically significant differences compared to SRR (M) in terms of measured slice similarities. Thus, SRR (S) and SRR (M) appear of similar volumetric self-consistency as quantified by the similarities between motion-corrected and respectively projected high-resolution volume slices.

**Fig. 18:**
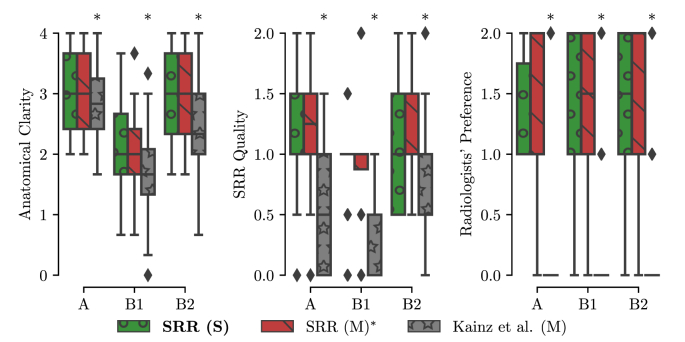
Summary of clinical evaluation. Two radiologists performed a qualitative assessment of the obtained high-resolution reconstructions regarding anatomical clarity, SRR quality and subjective preference involving 39 cases. A higher score indicates a better outcome. For anatomical clarity scores indicate how well CS, CAIF and LCF are visualized in each image with ratings 0 (structure not seen), 1 (poor depiction), 2 (suboptimal visualization; image not adequate for diagnostic purposes), 3 (clear visualization of structure but reduced tissue contrast; image-based diagnosis feasible), and 4 (excellent depiction; optimal for diagnostic purposes). SRR quality is a combined average score of individual visible artifacts and blur scores with ratings 0 (lots of artifacts/blur) to 2 (no artifact/blur). Radiologists' preference ranks subjectively from the least (0) to the most preferred (2) reconstruction. A * denotes a significant difference compared to SRR (M) based on a Wilcoxon signed-rank test (p<0.05). The results underline that SRR (M)/(S) represent high-quality reconstructions with high anatomical clarity that are visually indistinguishable and were subjectively preferred over Kainz et al. (M) by the two radiologists.

**Fig. 19:**
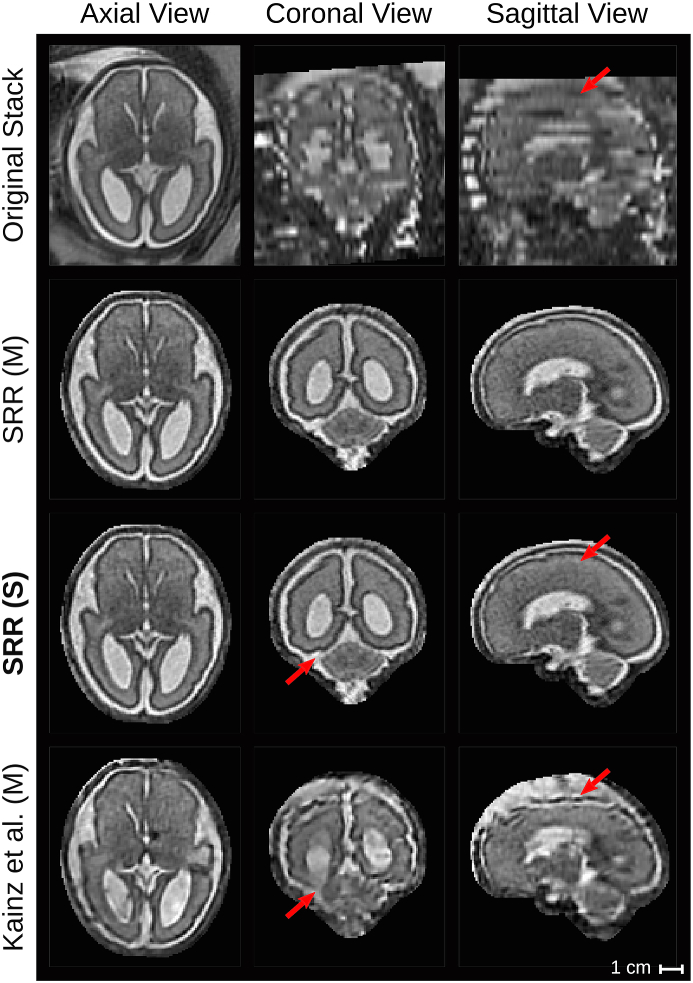
Qualitative comparison of reconstruction methods in the template space. The comparison shows the template space reconstructions of a group B2 subject (post-surgical SB, GA=27 weeks) based on 7 low-resolution input stacks. An original stack (linearly resampled) with resolution of $0.47^{2}\times 3$ mm^3^ is provided for reference. Red arrows show anatomical differences between SRR (S) and Kainz et al. (M).

**Fig. 20:**
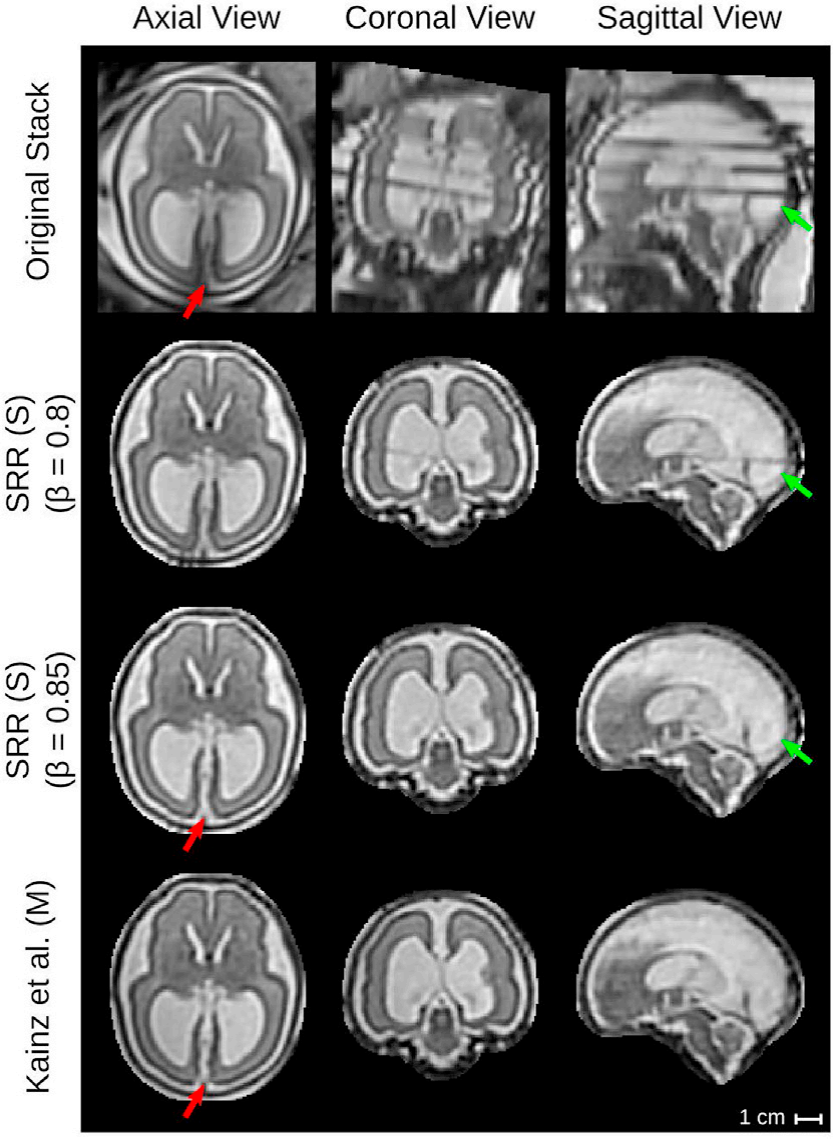
Qualitative comparison of reconstruction methods in the template space. The comparison shows the template space reconstructions of a group B2 subject (post-surgical SB, GA=26 weeks) based on 4 low-resolution input stacks. An original stack (linearly resampled) with resolution of $0.74^{2}\times 3$ mm^3^ is provided for reference. Green arrows indicate the rejection of the final intensity-artifacted slice of the original stack using the outlier-threshold β=0.85. Red arrows show anatomical differences between SRR (S) and Kainz et al. (M) in direct comparison with the original stack.

In absence of a ground-truth of the high-resolution volume, an additional subjective quality assessment in a clinical context was made. Two pediatric radiologists (MA and PP) assessed all reconstructions in the template-space side-by-side blinded to the reconstruction methods. The high-resolution masks obtained by SRR (M) were used for a consistent visual cropping of the reconstructions. The radiologists gave scores of three metrics on the results: 1) anatomical clarity of the cerebellar structure (CS), the cerebral aqueduct and the interhemispheric fissure (CAIF) and the longitudinal cerebral fissure (LCF) in the range of [0, 4], 2) SRR quality against introduced artifactual structures and edge uncertainty in the range of [0, 2], and 3) radiologists’ preference in the range of [0, 2]. A higher score for each metric indicates a better reconstruction. The evaluation results are summarized in Fig. 18 (a more detailed comparison of the individual scores is provided in the Inline Supplementary Figs. S9 and S10). It shows that SRR (S) and SRR (M) achieved high-quality reconstruction results that are subjectively almost indistinguishable. Moreover, both SRR (S) and SRR (M) were consistently preferred over Kainz et al. (M) by the radiologists which can be explained by the high anatomical clarity and SRR quality achieved by our proposed reconstruction framework. The comparison against Kainz et al. (M) confirms the effectiveness of our proposed outlier-robust SRR framework which is also illustrated in Fig. 14, Fig. 19, Fig. 20 and in the Inline Supplementary Figs. S6–S8.

**Fig. 21:**
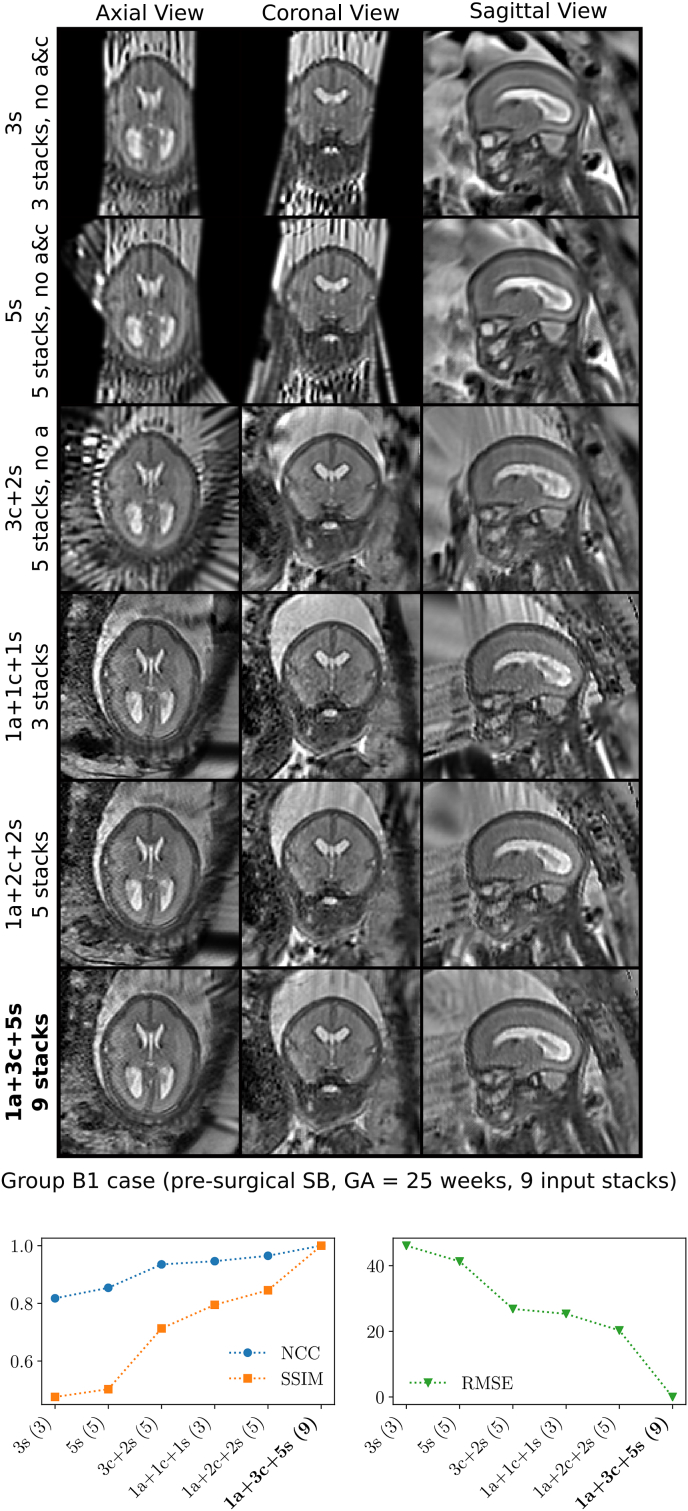
Comparison of obtained reconstructions in the template space for six different input data configurations using the case with the highest number of nine available input stacks (B1 subject, pre-surgical SB, GA=25 weeks). The horizontal axis for the quantitative comparisons is sorted in ascending order based on the NCC outcome, whereby “1a+3c+5s” constrained by its mask was used as reference. Using at least three stacks in three different orientations leads to a high anatomical detail in all three anatomical planes. Increasing the number of stacks per orientation can further increase the reconstruction quality. Additional comparisons for other cases are shown in Inline Supplementary Figs. S11 and S12.

For practical purposes, it is important to understand at which input data scenarios the proposed reconstruction framework can produce high-resolution 3D reconstructions with high anatomical fidelity. Using the case that is associated with the highest number of nine available input stacks, six different input data configurations were tested to evaluate the achievable reconstruction quality based on, (i) three approximately sagittal (“3s”, three stacks); (ii) five approximately sagittal (“5s”, five stacks); (iii) three approximately coronal and two approximately sagittal (“3c+2s”, five stacks); (iv) one approximately axial, one approximately coronal, and one approximately sagittal (“1a+1c+1s”, three stacks); (v) one approximately axial, two approximately coronal, and two approximately sagittal (“1a+2c+2c”, five stacks); and (vi) all available data, i.e. one approximately axial, three approximately coronal, and five approximately sagittal (“1a+3c+5s”, nine stacks). A qualitative comparison of the obtained template-space reconstructions using SRR (S) alongside the comparison of their quantitative similarity scores against “1a+3c+5s” (all data) based on NCC, SSIM and RMSE is provided in Fig. 21 (similar comparisons for other cases are shown in Inline Supplementary Figs. S11 and S12). For the quantitative evaluation, only masked voxels associated with the obtained high-resolution volume mask of “1a+3c+5s” were considered. The comparisons underline that at least three stacks in three different orientations are required in order to get reconstructions that show high anatomical detail in all three anatomical planes. In particular, the experiments illustrate that reconstructions based on three approximately orthogonal stacks can lead to better reconstructions compared to using five stacks acquired in only two orientations. Increasing the number of stacks per orientation can further increase the reconstruction quality.

Using our non-optimized implementation on a single computer with four CPUs, the typical processing time for SRR (S) was approximately 13min for the subject-space reconstruction, i.e. the computation of the two-step iterative motion-correction and volumetric reconstruction steps, and approximately 11min for the template-space reconstruction, i.e. the combined template-space alignment and volumetric reconstruction from motion-corrected slices. The reconstruction times for SRR (M) were comparable. For Kainz et al. (M) the subject-space reconstructions took approximately 6min on average.

## 5 Discussion and conclusion

Our automated pipeline for fetal brain reconstruction in MR imaging benefits from deep learning-based localization and segmentation where a CNN-based coarse segmentation is proposed for robust localization and a multi-scale loss function for a fine segmentation of the fetal brain. Compared with Keraudren et al. (2014), our localization method does not need prior information such as shape and size of the fetal brain and it achieved superior performance in less time. Different from Salehi et al. (2018), which takes a whole image as the input of a CNN, our segmentation method follows a coarse-to-fine approach. The benefit is three-fold: 1) it rejects false positives outside the localization result, 2) the training data for Seg-Net are local regions around the fetal brain and therefore they have less imbalance between the foreground and background, and 3) Seg-Net has lower memory requirements and is more efficient than working on the whole image. However, it requires training of two networks independently, and may be improved by joint training or adopting attention mechanisms (Oktay et al., 2018; He et al., 2017) in the future. Minimizing our proposed multi-scale loss function encourages a segmentation to be close to the ground truth at multiple non-local levels, and helps to obtain more spatially consistent results as shown in Fig. 9. Our testing set consisted of a relatively large dataset of 268 images characterized by a wide range of resolution and appearance differences. Results show that our localization and segmentation method is robust against images with bias field, different patient groups and gestational ages (Fig. 10, Fig. 11). However, it would be of interest to further investigate its ability to generalize to a wider range of MR acquisition parameters and gestational ages (e.g., fetuses in the third trimester).

Moreover, we present an alternative reconstruction framework that includes a novel outlier-rejection method for robust SRR. In contrast to Gholipour et al. (2010) and Kuklisova-Murgasova et al. (2012), our formulation leads to a simple, yet effective, linear least-squares problem with a single hyperparameter whose unique solution can be solved for very efficiently using dedicated least-squares solvers. Despite its simplicity, this outlier-rejection method using a single threshold parameter value was shown to allow for a remarkably robust elimination of outliers for most cases (Fig. 16, Fig. 20). We demonstrate that our proposed outlier-robust framework can produce high-quality high-resolution visualizations from highly heterogeneous and challenging clinical data with results superior to the state-of-the-art toolkit Kainz et al. (M) (Kainz et al., 2015b; Kuklisova-Murgasova et al., 2012). This involved stacks with multiple image resolutions per case including high slice thicknesses between 2.5 mm and 4.4 mm which can be severely affected by substantial motion and intensity artifacts. In particular, we show that high-fidelity reconstructions with clear tissue boundary definitions can be achieved even in case a corrupted target stack is selected (Fig. 14). Anecdotal evidence showed that the target stack selection method proposed by Kainz et al. (2015b), although slower, seems to be slightly better in choosing higher-quality stacks as initial reference than the automatic approach we presented. Even though our reconstruction framework showed good performance for our entire experimental dataset, a higher-quality initial reference may be beneficial for other cases in practice. To ease future work on this, it will be integrated in our open-source reconstruction software framework NiftyMIC.

Limitations of the comparison against Kainz et al. (M) include that the publicly available method^19^ only allows to specify a single mask for the target stack whereas our method can take advantage of using separate masks for all input stacks. This allows a more accurate motion correction for our method and can therefore lead to higher quality high-resolution reconstructions. A potential exclusion of heavy motion- or artifact-corrupted stacks is likely to improve the SRR quality further for cases that performed less satisfying (e.g. Inline Supplementary Fig. S8). An automatic exclusion criteria could be based on a motion score similar to the one presented in Kainz et al. (2015b). Or, more generally, a stack-specific automatic scoring system could be devised in addition to the outlier-rejection mechanism in order to prioritize stacks based on usability.

Additionally, we present a fast template space alignment method for high-resolution visualizations in the standard radiological anatomical planes that is robust to large brain morphology changes such as encountered in spina bifida. Further robustness to more substantial false-positives in the brain mask high-resolution volume (Fig. 13) could be achieved by using robust principal component analysis (Candès et al., 2011; Parikh and Boyd, 2014) to estimate the principal brain axes. Faster computational times for the high-resolution volume reconstructions can be achieved by more efficient multi-core or GPU implementations including an, in principle, trivially parallelizable computation of the, currently, sequentially performed rigid slice-to-volume registrations. In cases with a large inter-slice motion, the extracted bounding boxes will be larger than bounding boxes that tightly fit brains as captured on non-motion corruption stacks. This can lead to an increased computational time of the subsequent volumetric reconstruction step due to a larger region of interest provided to the algorithm. However, since we use the segmented brain mask for the reconstruction, the final reconstruction quality is less likely to be affected by this. Moreover, and as shown in our experiments (Fig. 14, Fig. 18), our method displays remarkable robustness even in such cases.

We investigated the impact of different input data configurations on our proposed automatic reconstruction pipeline. Based on these experiments, we conclude that at least three approximately orthogonal stacks are required for our SRR framework to obtain high-resolution reconstructions with high anatomical detail in all three anatomical planes. In particular, if only one or two orthogonal orientations are available, the obtained reconstruction quality is generally of inferior quality even if more than three stacks are used (Fig. 21). Using more stacks for each of the three (approximately) orthogonal orientations can further increase the reconstruction quality due to the improved recovery of partial voluming effects using the SRR formulation (5). Similar conclusions were drawn in Ebner et al. (2019) by performing controlled experiments in the context of super-resolution for upper abdominal SSFSE sequences. In our experience, at least five stacks in three approximately orthogonal orientations are desirable for fetal MRI. However, depending on the degree of motion corruption and image artifacts more stacks may be needed due to the potentially higher rate of required slice rejections.

Limitations of this study include that fetal MRI SSFSE sequences were acquired using two different scanners at a single imaging center. It would be of interest to investigate the performance of the proposed automatic segmentation and reconstruction method for fetal images using multiple scanners and imaging centers. In principle, however, the same volumetric reconstruction model appears promising for a range of other applications and anatomies provided a suitable parametrization of the PSF is available to define the slice acquisition model (4) for the used 2D MRI sequence. In particular, the outlier-robust reconstruction framework has shown good results also for other types of sequences such as upper abdominal single-shot T2-weighted (Ebner et al., 2019) and fetal resting-state functional MRI sequences (Sobotka et al., 2019). Similarly, the P-Net has also demonstrated good performance on other structures such as the placenta and the fetal lungs in our previous work (Wang et al., 2018). Beyond structural MRI, it would be of interest to test the applicability of our framework to obtain fully automated reconstructions for functional fetal MRI (Rutherford et al., 2019).

In conclusion, we present a fully automated, and publicly available,^18^ framework for fetal brain MRI localization, segmentation and super-resolution reconstruction. Our experiments with fetuses with normal brain anatomy as well as fetuses with brain changes associated with spina bifida show that the proposed pipeline produces automatic reconstructions that are comparable to manual segmentation-based reconstructions, therefore, effectively eliminating the need of any manual intervention. In the future, we aim to apply this framework to quantify the impact of spina bifida surgical closure by measuring the resolution of the Chiari type II malformation and the degree of ventriculomegaly.

## Declaration of competing interest

WL was employed by King's College London during most of the preparation of this work and was employed by the company Nvidia for the final editing and proofreading of the manuscript. SO is a founder and shareholder of BrainMiner Ltd, UK.

## Footnotes

2 The automatic localization and segmentation framework FETAL_BRAIN_SEG is available at https://github.com/gift-surg/fetal_brain_seg and is integrated in the outlier-robust SRR framework NiftyMIC (https://github.com/gift-surg/NiftyMIC).

3 http://crl.med.harvard.edu/research/fetal_brain_atlas.

4 https://www.tensorflow.org.

5 http://niftynet.io.

6 https://itk.org.

7 https://github.com/KCL-BMEIS/niftyreg.

8 https://github.com/Inria-Asclepios/SmoothingRecursiveYvvGaussianFilter.

9 https://github.com/gift-surg/ITK_NiftyMIC.

10 As we only correct for rigid brain motion, the surrounding maternal/fetal tissue reconstruction may be of limited anatomical accuracy.

11 https://github.com/gift-surg/fetal_brain_seg, version from 1 Feb 2019 was used.

12 https://github.com/gift-surg/NiftyMIC, version 0.7.3 was used.

13 https://github.com/BioMedIA/IRTK.

14 https://bitbucket.org/bchradiology/u-net/src.

15 https://github.com/bkainz/fetalReconstruction, version from 20 Jan 2017 was used.

16 For the reconstructions, we used the CPU version for both SVR and SRR as the GPU-accelerated approach tends to produce blurrier SRR outcomes in our experience. Therefore, the resulting benchmark method reduces, in principle, to the approach as described in Kuklisova-Murgasova et al. (2012). However, given that we use the implementation as provided by Kainz et al. (2015a) we stick to the notation “Kainz et al. (M)”.

17 https://fsl.fmrib.ox.ac.uk/fsl/fslwiki/FLIRT.

19 The automatic localization and segmentation framework FETAL_BRAIN_SEG is available at https://github.com/gift-surg/fetal_brain_seg and is integrated in the outlier-robust SRR framework NiftyMIC (https://github.com/gift-surg/NiftyMIC).

18 https://github.com/bkainz/fetalReconstruction.
