# Supplementary material for: An automated framework for localization, segmentation and super-resolution reconstruction of fetal brain MRI
Source: Neuroimage. 2020 Feb 1;206:116324. doi: 10.1016/j.neuroimage.2019.116324 (PMC7103783; doi:10.1016/j.neuroimage.2019.116324)
Supplement: Multimedia component 1 [file mmc1.pdf]

Supplementary Material

An Automated Framework for Localization, Segmentation and Super-Resolution Reconstruction of Fetal Brain MRI

Michael Ebner*, Guotai Wang*, Wenqi Li, Michael Aertsen, Premal A. Patel, Rosalind Aughwane, Andrew Melbourne, Tom Doel, Steven Dymarkowski, Paolo De Coppi, Anna L. David, Jan Deprest, Sébastien Ourselin, Tom Vercauteren

* Authors contributed equally

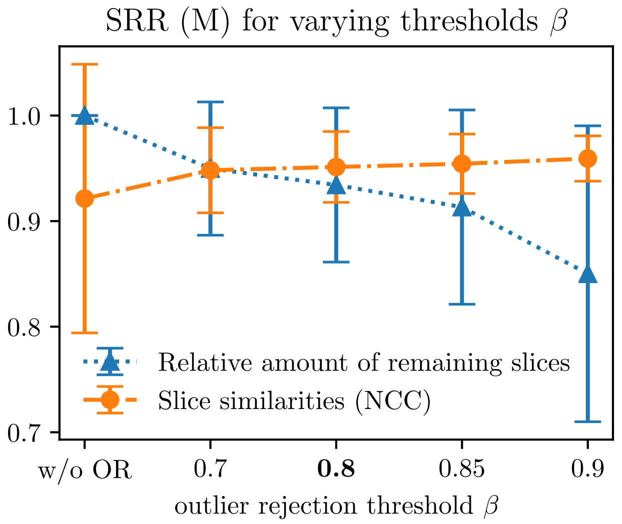

Figure S1: Impact of outlier threshold on slice retention and slice similarities. Impact of outlier rejection threshold β on the slice similarities $\text{Sim}(\mathbf{y}_k^i, \mathbf{A}_k^i\mathbf{x}^i)$ for SRR (M) for the respectively remaining $\mathcal{K}_\beta^i$-slices at iteration $i = 3$. The error bars indicate the mean and standard deviation. A good balance between a high number of retained slices and high slice similarity (indicating a good self-consistency of the obtained SRR) appears to be around $\beta = 0.8$.

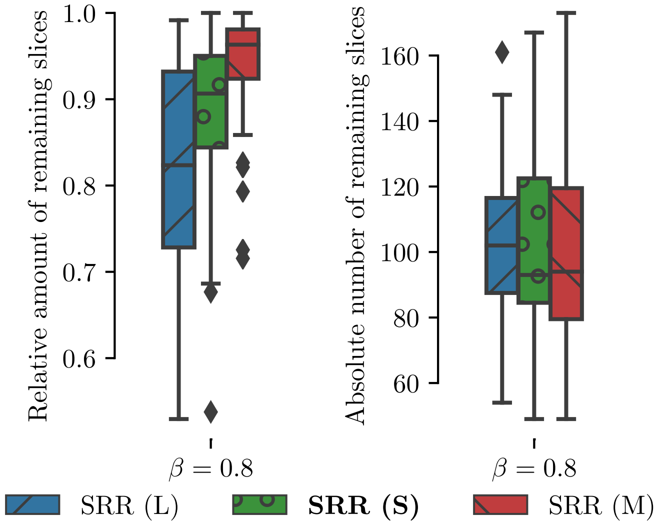

Figure S2: Impact of outlier threshold for different inputs masks. Comparison of remaining slices given by $\mathcal{K}_\beta^i$ at iteration $i = 3$ for the outlier-robust SRR algorithm using different input masks. Automatic segmentations lead to a higher rate of slice rejections compared to using the manual input masks but result in an overall comparable number of remaining slices. Thus, slices with false-positive segmentations are automatically detected and rejected by the SRR algorithm.

Sensitivity of SRR algorithm to Outlier-threshold and Input Masks

Experiments were performed to investigate the sensitivity of the proposed high-resolution reconstruction method to the outlier-threshold β and the input fetal brain masks. Fig. S1 shows how the outlier-threshold β impacts the number of retained slices used for solving the SRR problem (5). The higher β the higher and less variant the volumetric self-consistency becomes as expressed by the slice similarities $\text{Sim}(\mathbf{y}_k^i, \mathbf{A}_k^i\mathbf{x}^i)$ after the final iteration $i = 3$. However, the number of slice rejections substantially increases beyond $\beta = 0.8$ whereas the NCC of measured slice similarities plateaus. To strike a balance between conservative slice retention and effective outlier rejection we select $\beta = 0.8$ as the defining outlier-threshold for our method. Fig. S2 illustrates the slice rejection performance based on different input masks. In case of manual masks, typically well beyond 90 % of all slices are retained for the volumetric reconstruction. Automatically obtained masks lead to a higher rate of slice rejections but result in an overall comparable number of remaining slices for the SRR step. Thus, slices with false-positives segmentations are automatically detected and rejected by the SRR algorithm.

Convergence of Motion Correction Estimates for Iterative Two-Step Algorithm

Fig. S3 shows the convergence of estimated motion correction parameters for each registration step within the two-step motion-correction/volumetric reconstruction cycle. In particular, it shows that after the third slice-to-volume registration (SVR) step, parameter changes become negligible,

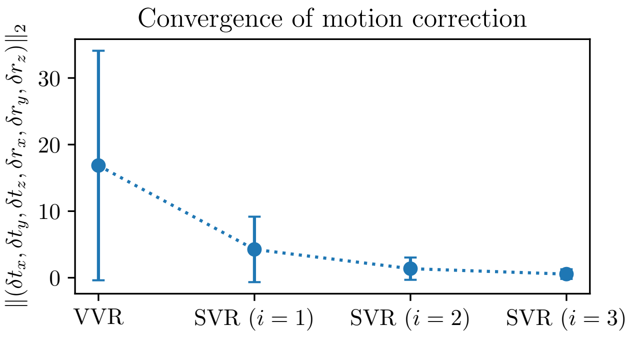

Figure S3: Convergence of motion correction parameters for the two-step iterative SRR algorithm. Mean and standard deviation of the ℓ^2-norm of incremental translation δt_x, δt_y, δt_z (mm) and rotation δr_x, δr_y, δr_z (degree) parameters are shown for the volume-to-volume registration (VVR) and the individual slice-to-volume registration (SVR) iteration (i) steps for all 39 cases based on SRR (S). After three two-step motion-correction/volumetric-reconstruction cycles, parameter changes become negligible.

therefore suggesting convergence of the two-step motion-correction/volumetric-reconstruction algorithm at this point.

Assessment of Intensity Correction Steps

To investigate the influence of the proposed intensity correction steps on the reconstruction results, SRR (S) outcomes are computed for four intensity correction scenarios: (i) no intensity correction of individual stacks (”Original”), (ii) bias field correction (N4ITK, Tustison et al. (2010)) for all stacks (”BFC”), (iii) intensity correction using linear regression with target stack reference values (”IC”), and (iv) IC following BFC (”IC$\circ$ BFC”) as the proposed method. Based on each configuration, all SRR (S) outcomes were computed and compared in the template space whereby the SRR template-space outcome of IC $\circ$ BFC was chosen for template-space alignment for all the remaining outcomes. Quantitative comparisons were performed using NCC, SSIM and RMSE whereby only the masked voxels associated with the high-resolution mask obtained by the manual-mask reconstruction SRR (M) outcome was used for evaluation (Fig. S4). All reconstructions were successful for all 39 cases regardless of the intensity correction scenario, which is also indicated by the consistently high absolute similarity values suggesting little sensitivity of SRR (S) to the performed intensity correction steps. This observed robustness of motion correction against image intensity changes can in part be explained by the choice of NCC as similarity measure for the registration steps, which is insensitive to the performed linear intensity correction (IC). With respect to the overall reconstruction quality, however, statistical tests based on Kruskal-Wallis and post hoc Dunn's tests indicate that both the bias field correction and subsequent linear intensity correction steps lead to statistically significant improvements towards more coherent intensity values of the obtained volumetric reconstructions.

Volume-matched vs Age-matched Template Space Alignment

The success rate of the proposed template-space alignment approach, i.e principal brain axes (PBA)-initialized block-matching registration, was tested for different template selections from the spatiotemporal atlas (Gholipour et al., 2017). Four different template space selections were considered: (i) volume-matched template (proposed), (ii) gestational age-matched template (GA, in weeks) as proposed in (Tourbier et al., 2017), (iii) template corresponding to one week older than gestational age-matched template (GA + 1), and (iv) template corresponding to one week younger than gestational age-matched template (GA − 1). A template space alignment was considered successful if a correct alignment in the standard anatomical planes was confirmed visually. Table S1 illustrates that the proposed volume-matched template selection leads to more robust template-space alignments compared to using the age-matched template. By systematically underestimating the gestational age of the template by one week, the same success rate can be achieved. This supports the argument that brains affected by spina bifida appear typically smaller than normal brains of the same age. Importantly, however, the high success rates across actual template selections highlight the high robustness of the proposed template-space alignment approach based on PBA-initialized block-matching.

Table S1: Comparison of template-space alignment success rates of the proposed template space alignment approach based on using the volume-matched template (proposed), the gestational age-matched template (GA, in weeks), and the templates associated with one week older and younger than GA.

	SRR (S)			SRR (M)		
	A	B1	B2	A	B1	B2
GA + 1	**7**	13	14	**7**	14	**16**
GA	**7**	13	**15**	**7**	**16**	**16**
GA − 1	**7**	**15**	**15**	**7**	**16**	**16**
**volume-matched**	**7**	**15**	**15**	**7**	**16**	**16**
Total number of cases	7	16	16	7	16	16

Assessment of Volumetric Self-Consistency

In addition to the performed slice similarity comparisons $\text{Sim}(\mathbf{y}_k^i, \mathbf{A}_k^i\mathbf{x}^i)$ after the final SVR-SRR iteration ($i = 3$) using SSIM and PSNR in Main Manuscript Fig. 17, we also performed comparisons using NCC, normalized mutual information (NMI), root mean squared error (RMSE) and mean absolute error (MAE). Fig. S5 summarizes the comparisons which corroborate the findings using SSIM and PSNR that SRR (S)/(M) appear of similar volumetric self-consistency.

Extended Qualitative Comparison of Reconstruction Methods

Additional visual comparisons of the obtained high-resolution reconstructions are provided in Figs. S6 to S8 as extension to the ones in Main Manuscript Figs. 14, 19 and 20 to illustrate the effectiveness of our proposed outlier-robust SRR framework. In particular, it shows the ability to reconstruct clear tissue boundaries with high anatomical accuracy even in case of challenging, artifact-corrupted input data.

Extended Clinical Evaluation

Figs. S9 and S10 represent an extension to Main Manuscript Fig. 18 and provide a more detailed comparison of the individual scores regarding anatomical clarity and SRR quality.

Extended Comparison of SRR Quality vs Input Data

Figs. S11 and S12 provide additional comparisons to Main Manuscript Fig. 21 to show obtained high-resolution 3D reconstructions for different input data scenarios.

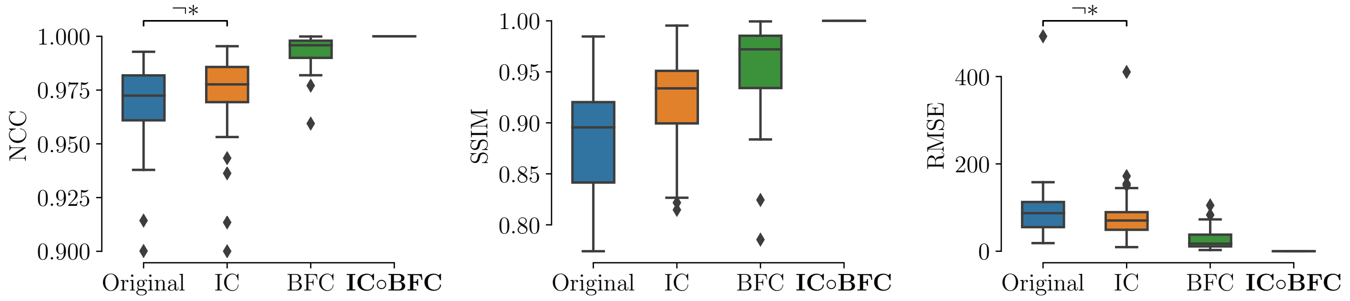

Figure S4: Quantitative comparison of the obtained SRR (S) outcomes in the template space for all 39 cases using no intensity correction ("Original"), bias field correction ("BFC"), and linear intensity correction ("IC") against the outcome of the proposed approach, i.e. IC following BFC ("IC∘ BFC"). All configurations for the SRR (S) outcomes apart from Original vs. IC are statistically significant based on Kruskal-Wallis with post hoc Dunn's tests for all evaluated similarity measures.

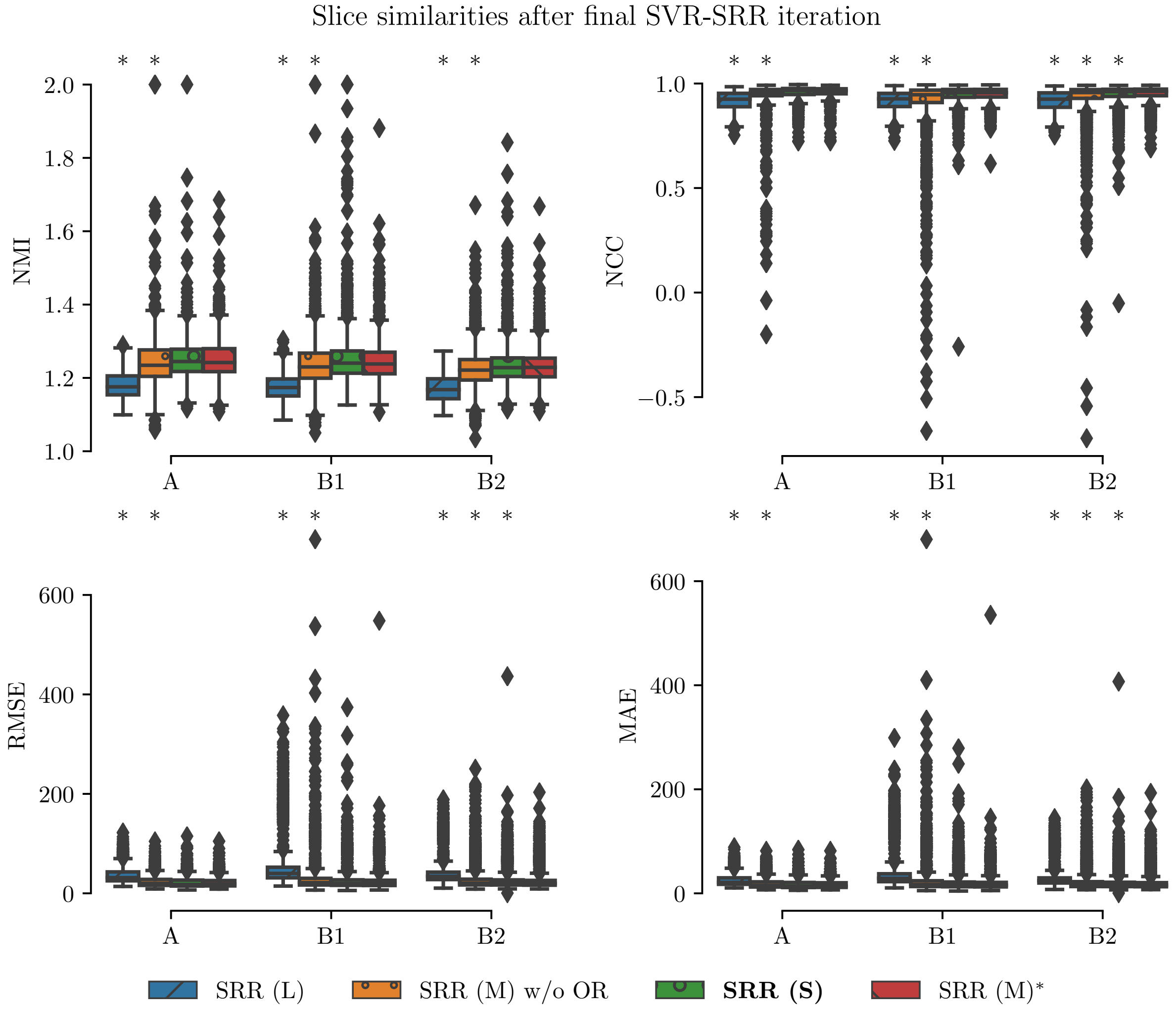

Figure S5: Slice similarities after the final SVR-SRR iteration. Quantitative comparison of different reconstruction methods based on $\mathrm{Sim}(\mathbf{y}_k^i, \mathbf{A}_k^i \mathbf{x}^i)$ after the final SVR-SRR iteration ($i = 3$). A * denotes a significant difference compared to SRR (M) within each group based on Kruskal-Wallis with post-hoc Dunn tests ($p < 0.05$). Thus, SRR (S) and SRR (M) appear of similar volumetric self-consistency as quantified by the similarities between motion-corrected and respectively projected high-resolution volume slices.

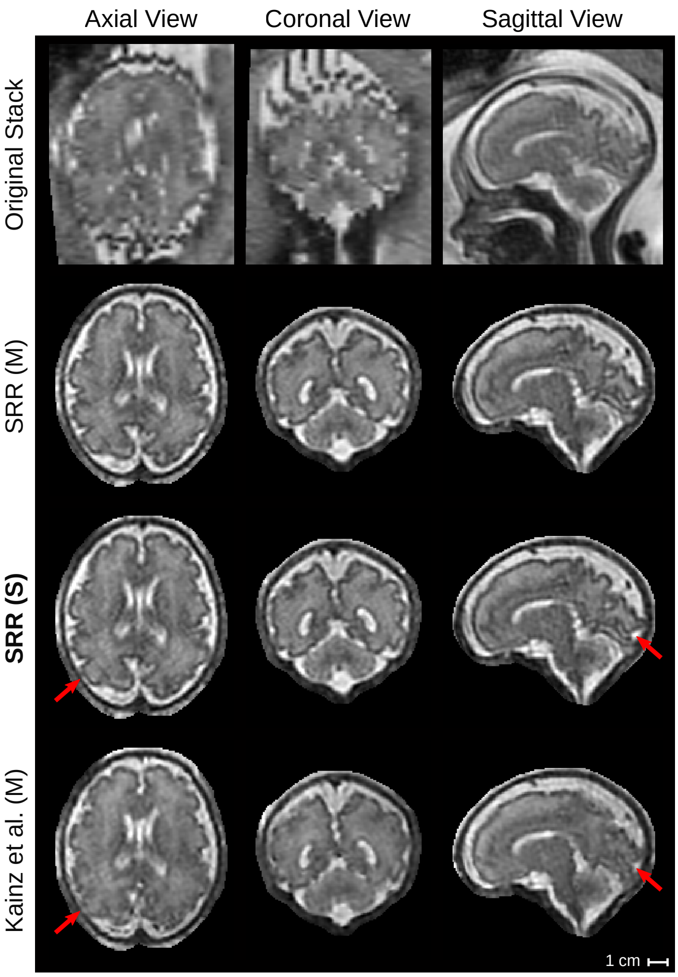

Figure S6: Qualitative comparison of reconstruction methods in the template space. The comparison shows the template space reconstructions of a group A subject (normal, GA = 30 weeks) based on 7 low-resolution input stacks (subject space SRRs are shown in Main Manuscript Fig. 14b). An original stack (linearly resampled) with resolution of $0.74^2 \times 3$ mm^3 is provided for reference. Red arrows indicate example differences in the reconstruction outcomes compared to Kainz et al. (M).

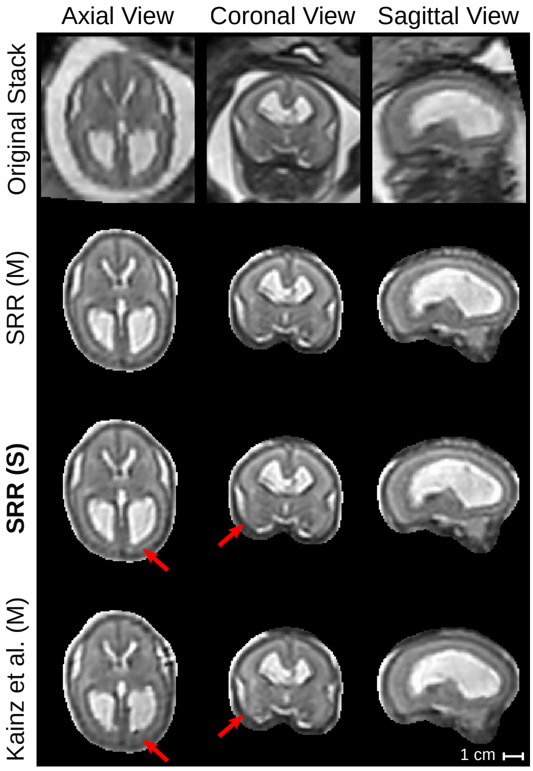

Figure S7: Qualitative comparison of reconstruction methods in the template space. The comparison shows the template space reconstructions of a Group B1 subject (pre-surgical SB, GA = 23 weeks) based on 6 low-resolution input stacks. An original stack (linearly resampled) with resolution of $0.74^2 \times 3$ mm^3 is provided for reference. Red arrows show differences between SRR (S) and Kainz et al. (M).

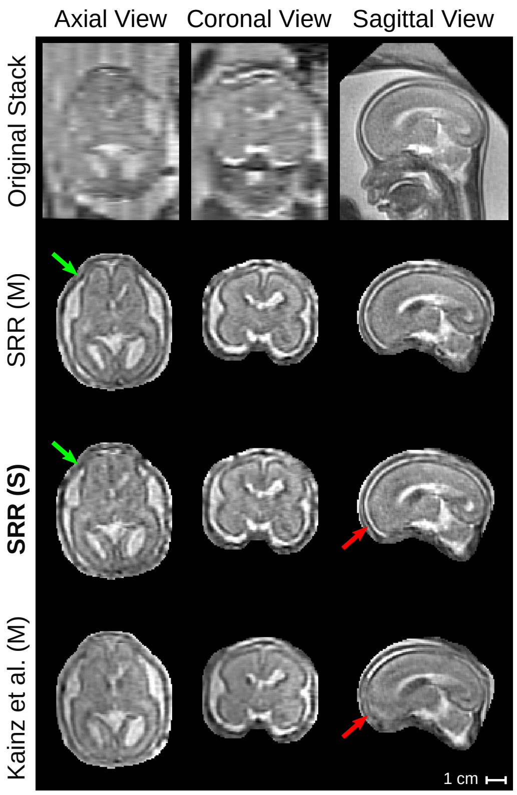

Figure S8: Qualitative comparison of reconstruction methods in the template space. The comparison shows the template space reconstructions of a group B1 subject (pre-surgical SB, GA = 23 weeks) based on 6 low-resolution input stacks. An original stack (linearly resampled) with resolution of $0.39^2 \times 4$ mm^3 is provided for reference. It represents the only case where SRR (M) is markedly better than SRR (S). Green arrows indicate differences between SRR (M) and SRR (S). Red arrows show differences between SRR (S) and Kainz et al. (M).

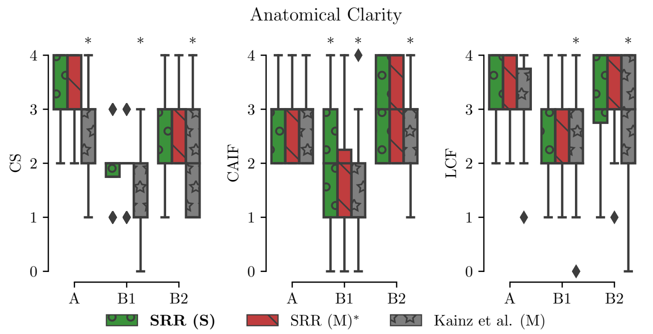

Figure S9: Summary of clinical evaluation for anatomical clarity scores. Two radiologists performed a qualitative assessment of the obtained high-resolution reconstructions regarding anatomical clarity involving 39 cases. Scores indicate how well cerebellar structure (CS), cerebral aqueduct and interhemispheric fissure (CAIF) and longitudinal cerebral fissure (LCF) are visualized in each image with ratings 0 (structure not seen), 1 (poor depiction), 2 (suboptimal visualization; image not adequate for diagnostic purposes), 3 (clear visualization of structure but reduced tissue contrast; image-based diagnosis feasible), and 4 (excellent depiction; optimal for diagnostic purposes). A * denotes a significant difference compared to SRR (M) based on a Wilcoxon signed-rank test ($p < 0.05$).

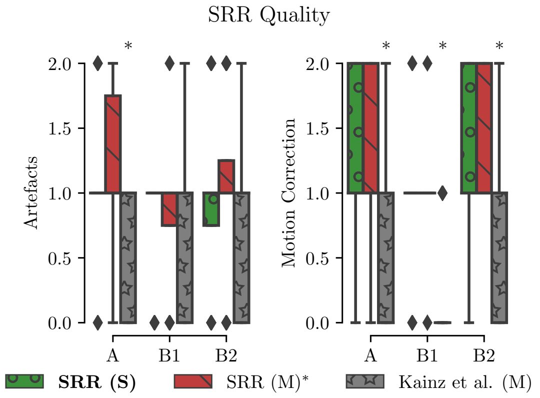

Figure S10: Summary of clinical evaluation for SRR quality scores. Two radiologists performed a qualitative assessment of the obtained high-resolution reconstructions regarding SRR quality involving 39 cases. The SRR quality was described by the visible artifacts and blur scores with ratings 0 (lots of artifacts/blur) to 2 (no artifact/blur). A * denotes a significant difference compared to SRR (M) based on a Wilcoxon signed-rank test ($p < 0.05$).

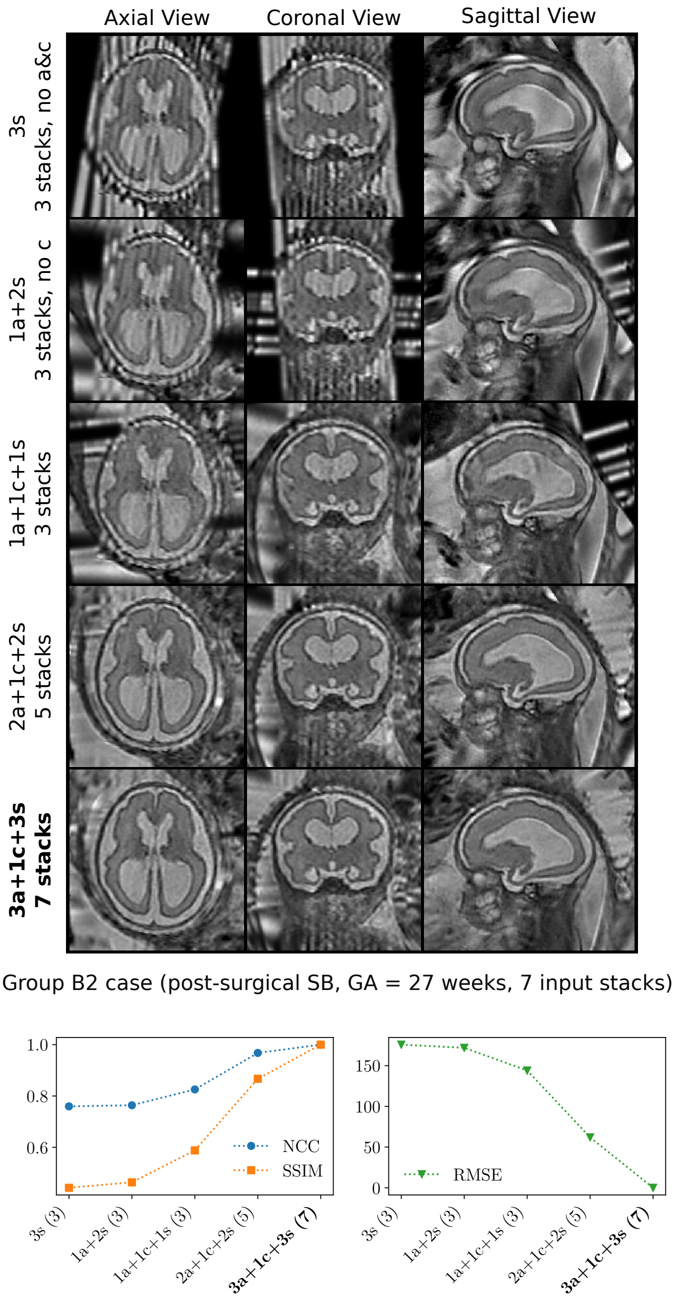

Figure S11: Comparison of obtained reconstructions in the template space for six different input data configurations using the case with the highest number of nine available input stacks (B2 subject, post-surgical SB, GA = 27 weeks). The horizontal axis for the quantitative comparisons is sorted in ascending order based on the NCC outcome, whereby ”3a+1c+3s” constrained by its mask was used as reference. Due to a high rate of axial slice rejections for ”1a+1c+1s” more than these three approximately orthogonal stacks are needed to achieve a high anatomical detail in all three anatomical planes.

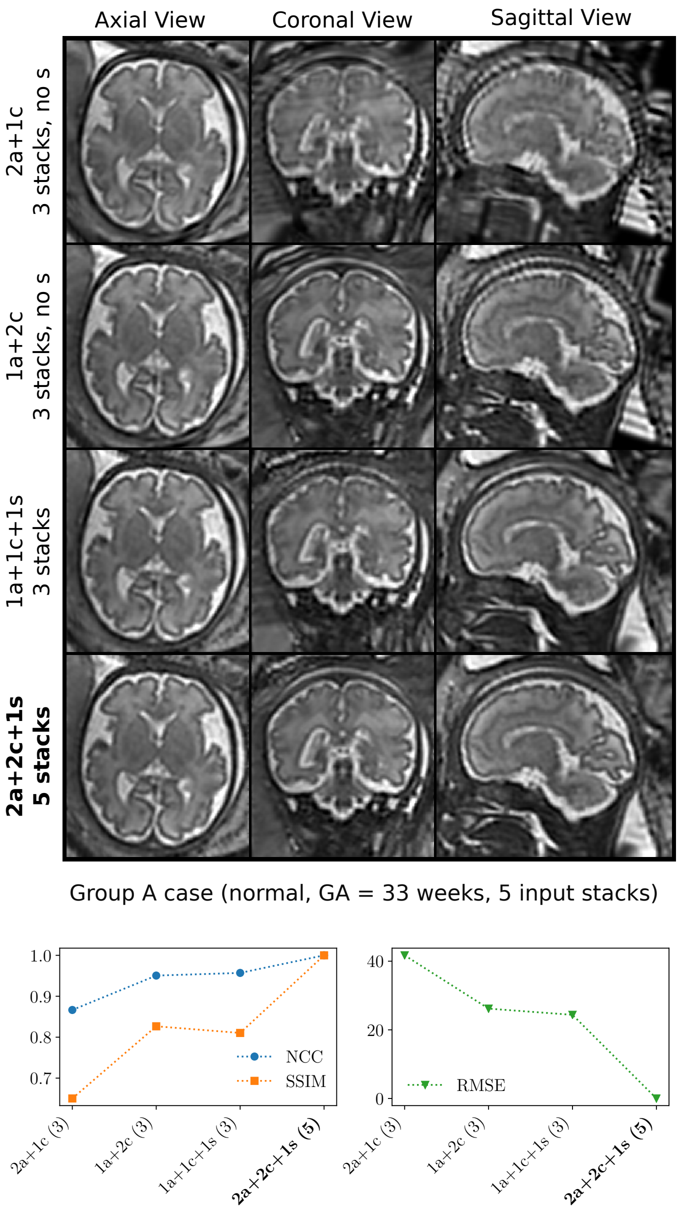

Figure S12: Comparison of obtained reconstructions in the template space for six different input data configurations using the case with the highest number of nine available input stacks (A subject, normal, GA = 33 weeks). The horizontal axis for the quantitative comparisons is sorted in ascending order based on the NCC outcome, whereby ”2a+2c+1s” constrained by its mask was used as reference. Using at least three stacks in three different orientations leads to a high anatomical detail in all three anatomical planes. Increasing the number of stacks per orientation can further increase the reconstruction quality.

References

Gholipour, A., Rollins, C. K., Velasco-Annis, C., Ouaalam, A., Akhondi-Asl, A., Afacan, O., Ortinau, C. M., Clancy, S., Limperopoulos, C., Yang, E., Estroff, J. A., and Warfield, S. K. (2017). A normative spatiotemporal MRI atlas of the fetal brain for automatic segmentation and analysis of early brain growth. *Scientific Reports*, 7(1):476.

Tourbier, S., Velasco-Annis, C., Taimouri, V., Hagmann, P., Meuli, R., Warfield, S. K., Bach Cuadra, M., and Gholipour, A. (2017). Automated template-based brain localization and extraction for fetal brain MRI reconstruction. *NeuroImage*, 155:460–472.

Tustison, N. J., Avants, B. B., Cook, P. A., Zheng, Y., Egan, A., Yushkevich, P. A., and Gee, J. C. (2010). N4ITK: Improved N3 Bias Correction. *IEEE Transactions on Medical Imaging*, 29(6):1310–1320.
